# Perception and Localization Error Propagation and Robust Path-Tracking Control for Agricultural Machinery in Hilly Orchards: A Review

**DOI:** 10.3390/s26154940

**Published:** 2026-08-04

**Authors:** Zhenlei Zhang, Yunfei Wang, Hanquan Lei, Xiang Dong, Weidong Jia

**Affiliations:** 1 School of Agricultural Engineering, Jiangsu University, Zhenjiang 212013, China; 2222416001@stmail.ujs.edu.cn (Z.Z.); 2112416035@stmail.ujs.edu.cn (Y.W.); 2222516027@stmail.ujs.edu.cn (H.L.); dongxiang@ujs.edu.cn (X.D.); 2 High-Tech Key Laboratory of Agricultural Equipment and Intelligence of Jiangsu Province, Jiangsu University, Zhenjiang 212013, China

**Keywords:** hilly orchards, perception and localization errors, reference path generation, path-tracking control, robust control

## Abstract

Hilly orchards are characterized by undulating terrain, canopy occlusion, irregular tree-row structures, and marked variations in ground adhesion conditions, which challenge the autonomous navigation of agricultural machinery by degrading perception and localization, increasing reference path uncertainty, and reducing closed-loop robustness. Perception and localization errors can propagate progressively along the chain of “sensor observation–vehicle pose estimation/environmental-structure perception–reference path generation–path-tracking controller inputs–vehicle closed-loop response,” ultimately affecting path-tracking accuracy, control smoothness, and operational safety. This review examines perception and localization error propagation and robust path-tracking control for agricultural machinery in hilly orchards. The review first summarizes the navigation roles and error characteristics of key observation sources and then analyzes how perception, localization, and reference path generation provide state variables, reference variables, and safety constraints to controllers. It subsequently compares typical path-tracking methods under multi-source disturbances and actuator constraints and discusses key challenges and an integrated robust design direction coupling perception and localization, reference path generation, and path-tracking control. The proposed integrated framework represents a synthesis-derived design direction rather than a complete architecture that has already been experimentally validated in hilly orchard field environments.

## 1. Introduction

With the development of precision agriculture, smart orchards, and autonomous agricultural equipment, orchard production is shifting from labor-intensive, experience-based management toward an efficient, precise, and autonomous operation mode [1,2,3,4,5,6]. Orchard agricultural machinery and mobile robots have been increasingly applied to fertilization, spraying [7], weeding, transportation, inspection, and harvest assistance [8,9,10], among which path-tracking control, as the closed-loop execution component of autonomous navigation systems, is a key technology for ensuring stable driving, precise operation, and continuous operation of agricultural machinery [11,12,13]. Compared with flat farmland and regularly structured orchards, hilly orchards are characterized by uneven and undulating terrain, with representative slope angles reaching approximately 5–15° [14], as well as canopy occlusion, irregular tree rows, narrow inter-row spaces, and marked variations in ground adhesion conditions. In this review, longitudinal slope refers to the terrain inclination along the direction of travel and mainly affects traction demand, longitudinal slip, drive requirements, and reference-speed selection, whereas cross-slope refers to the terrain inclination perpendicular to the direction of travel and mainly affects sideslip, roll stability, heading deviation, and safety constraints. These complex environmental factors mean that autonomous navigation of agricultural machinery is no longer a problem that can be independently solved by a single localization, path-planning, or control algorithm, but rather a system-level problem formed by the coupling of perception and localization, environmental-structure understanding, reference path generation, and closed-loop path tracking [15,16,17,18,19].

Perception and localization errors are an important source of degradation in path-tracking performance [20,21]. The unstructured environment of hilly orchards first degrades the quality of sensor observations and further affects vehicle pose estimation, environmental-structure perception, and reference path generation [22,23,24]. Subsequently, these errors do not directly manifest as controller failure, but instead propagate progressively along the chain of “sensor observation–vehicle pose estimation/environmental-structure perception–reference path generation–path-tracking controller inputs–vehicle closed-loop response” [25,26]. Specifically, inaccurate vehicle pose estimation may cause the controller to misjudge the actual motion state of agricultural machinery, environmental-structure perception errors may lead to instability in the reference driving direction, and slope-induced slip, sideslip, and variations in adhesion conditions may weaken the consistency between control commands and the actual vehicle response [27]. Therefore, the difficulty of path-tracking control in hilly orchards lies not merely in improving the tracking accuracy of a specific control algorithm, but in systematically revealing how complex environmental disturbances induce perception and localization errors, how these errors are transformed into deviations in control information, and how they ultimately affect closed-loop path-tracking performance [28,29].

In recent years, autonomous navigation of orchard mobile robots has developed a substantial body of research [30,31,32,33]. Existing reviews generally organize the literature by system components or algorithm categories to summarize sensor performance, path-planning algorithms, and path-tracking control strategies, or focus on comparing the applicable scenarios and technical characteristics of different control methods [34,35,36]. However, existing reviews still have the following limitations [37]. First, related studies have mostly been organized within a modular framework of “perception and localization–path planning–motion control,” with limited systematic analysis of how front-end sensor observation errors and vehicle pose estimation errors are further transformed into deviations in controller inputs, such as lateral error, heading error, reference curvature, reference speed, and safety constraints. Second, existing reviews on orchard navigation have mainly focused on flat or regularly structured orchard environments, while the error propagation mechanisms caused by slope-induced slip, cross-slope-induced sideslip, canopy occlusion, global navigation satellite system (GNSS) signal blockage and multipath effects, irregular tree rows, and reference path uncertainty in hilly orchards remain insufficiently discussed [38,39,40]. Third, existing reviews on path-tracking control have mostly emphasized performance comparisons among algorithms themselves, while relatively little attention has been paid to the influence of uncertainty in controller inputs on closed-loop tracking performance from the perspectives of perception confidence, reference path quality, vehicle slip state, and actuator constraints [41].

**Figure 1:**
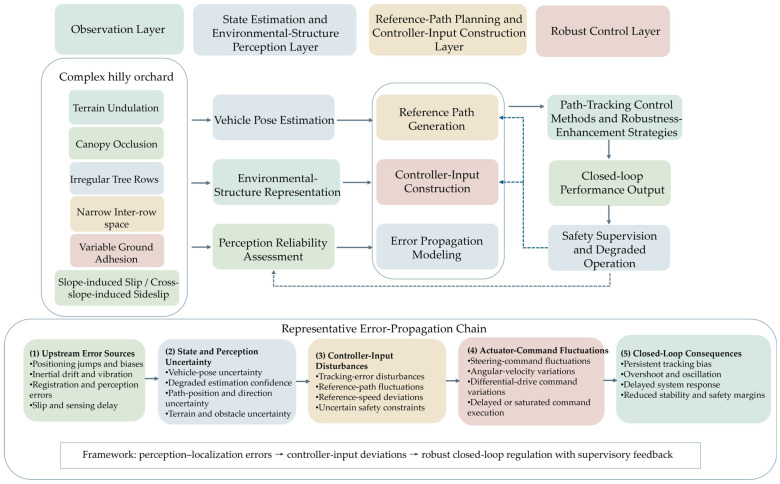
Overall framework of perception and localization error propagation and robust path-tracking control for agricultural machinery in hilly orchards.

Therefore, it is necessary to systematically review the key technologies of perception and localization, reference path generation, and path-tracking control for agricultural machinery in hilly orchards, with “how perception and localization errors affect path-tracking control” as the main thread. To address the above limitations, this review examines perception and localization error propagation and robust closed-loop path-tracking control for agricultural machinery under environmental disturbances in hilly orchards, as illustrated in Figure 1. First, an analytical thread is established for the propagation of perception and localization errors to path-tracking controller inputs in agricultural machinery operating in hilly orchards, revealing the influence of sensor observation errors, pose estimation errors, and environmental-structure perception errors on controller inputs such as lateral error, heading error, reference curvature, and reference speed; second, the roles of multi-source observation, fusion-based localization, environmental-structure representation, and reference path generation in constructing controller inputs are summarized, and their effects on the reliability of state feedback, reference paths, and safety constraints under complex disturbances in hilly orchards are analyzed; finally, the applicability and robustness-enhancement directions of typical path-tracking control methods are compared under conditions of slope-induced slip, cross-slope-induced sideslip, actuator constraints, and perception uncertainty, providing a reference for the integrated design of perception and localization, reference path generation, and path-tracking control for agricultural machinery in hilly orchards. The distinctive contribution of this review lies in its four-layer, controller-oriented decomposition of the navigation chain, its explicit mapping of perception and localization errors to controller-input disturbances and closed-loop consequences, and its full-chain robust adaptation framework incorporating perception reliability, reference path quality, vehicle–ground interaction, actuator constraints, and safety supervision.

### Review of Methodology

To ensure the relevance and representativeness of the reviewed literature, this study adopted a structured literature-search and screening approach. The Web of Science Core Collection, Scopus, and IEEE Xplore were systematically searched for relevant studies published from 2002 to July 2026, with the final search conducted on 31 July 2026. The searches yielded 196 records from the Web of Science Core Collection, 178 records from Scopus, and 82 records from IEEE Xplore. An additional 12 records were identified by screening the reference lists of relevant reviews and key studies. In total, 468 records were identified before duplicate removal.

Boolean operators were used to construct the search queries by integrating the following core concepts:

Subject terms: “orchard,” “hilly orchard,” “sloped orchard,” “terraced orchard,” “agricultural machinery,” and “orchard robot.”

Key technology terms: “GNSS,” “IMU,” “LiDAR,” “vision,” “localization,” “sensor fusion,” “SLAM,” “tree-row detection,” and “reference path.”

Algorithmic terms: “path tracking,” “trajectory tracking,” “Pure Pursuit,” “Stanley,” “PID,” “model predictive control,” “sliding-mode control,” and “robust control.”

The search syntax was adapted to the field requirements of each database while retaining the same conceptual combination of orchard or agricultural-machine environments and autonomous navigation technologies. The complete database-specific search strings were as follows.

Web of Science Core Collection: TS = ((“orchard” OR “hilly orchard” OR “sloped orchard” OR “terraced orchard” OR “agricultural machinery” OR “orchard robot”) AND (“GNSS” OR “IMU” OR “LiDAR” OR “vision” OR “localization” OR “sensor fusion” OR “SLAM” OR “tree-row detection” OR “reference path” OR “path tracking” OR “trajectory tracking” OR “Pure Pursuit” OR “Stanley” OR “PID” OR “model predictive control” OR “sliding-mode control” OR “robust control”)).

Scopus: TITLE-ABS-KEY ((“orchard” OR “hilly orchard” OR “sloped orchard” OR “terraced orchard” OR “agricultural machinery” OR “orchard robot”) AND (“GNSS” OR “IMU” OR “LiDAR” OR “vision” OR “localization” OR “sensor fusion” OR “SLAM” OR “tree-row detection” OR “reference path” OR “path tracking” OR “trajectory tracking” OR “Pure Pursuit” OR “Stanley” OR “PID” OR “model predictive control” OR “sliding-mode control” OR “robust control”)).

IEEE Xplore, All Metadata: ((“orchard” OR “hilly orchard” OR “sloped orchard” OR “terraced orchard” OR “agricultural machinery” OR “orchard robot”) AND (“GNSS” OR “IMU” OR “LiDAR” OR “vision” OR “localization” OR “sensor fusion” OR “SLAM” OR “tree-row detection” OR “reference path” OR “path tracking” OR “trajectory tracking” OR “Pure Pursuit” OR “Stanley” OR “PID” OR “model predictive control” OR “sliding-mode control” OR “robust control”)).

To focus on perception and localization error propagation and robust path-tracking control for agricultural machinery in hilly orchards, the following inclusion criteria were established:

1. The study addressed at least one core stage of autonomous navigation, including sensor observation, vehicle-state estimation, environmental-structure perception, reference path and controller-input construction, or path-tracking control.
2. The study concerned orchard machinery, orchard robots, or agricultural ground vehicles operating in directly relevant agricultural environments.
3. The study involved algorithm development, system integration, simulation validation, laboratory experiments, and field experiments.
4. Eligible publication types included peer-reviewed journal articles and conference papers.

All authors participated in the literature screening, and disagreements were resolved through discussion to ensure consistency. The screening process was conducted as follows:

1. All retrieved records were imported into EndNote 20 (Clarivate, Philadelphia, PA, USA) for duplicate removal, followed by an initial screening of titles and abstracts.
2. The full texts of studies that passed the initial screening were assessed according to the predefined inclusion and exclusion criteria. Studies focusing exclusively on fruit detection, disease diagnosis, yield estimation, or harvesting-target recognition without a navigation-related component were excluded.
3. The reference lists of relevant reviews and key studies were examined to identify important publications that might have been missed during the database searches.

To distinguish the strength of terrain-specific evidence, the included studies were classified as strong direct evidence from field experiments conducted in explicitly hilly or sloped orchards; limited direct evidence from hilly orchard simulations or studies explicitly described as hilly or sloped but without quantified slope information; indirect evidence from flat or regularly structured orchards and transferable agricultural environments; or as general methodological evidence. Studies without explicit terrain descriptions or reported terrain information were not treated as direct evidence of performance under hilly orchard conditions.

After the removal of 121 duplicate records, 347 unique records underwent title-and-abstract screening, of which 116 were excluded because they were outside the scope of autonomous navigation or insufficiently related to sensor observation, perception and localization, reference path generation, or path-tracking control. The full texts of the remaining 231 publications were assessed according to the predefined eligibility criteria, and a further 32 publications were excluded because they focused exclusively on fruit detection, disease diagnosis, yield estimation, or harvesting-target recognition without a navigation-related component, or because they were insufficiently relevant to the technical chain examined in this review. Finally, 199 publications were included in the qualitative synthesis. Analysis of the resulting literature base showed that previous reviews have generally focused on individual modules, such as sensing, localization, path planning, or control algorithms, and have mainly addressed flat farmland or regularly structured orchard environments. Few studies have systematically integrated error-propagation mechanisms and robust control methods across the complete technical chain of “sensor observation–state estimation and environmental-structure perception–reference path and controller-input construction–path-tracking control–vehicle closed-loop response” under complex hilly orchard constraints, including terrain undulation, canopy occlusion, slip, and sideslip.

**Figure 2:**
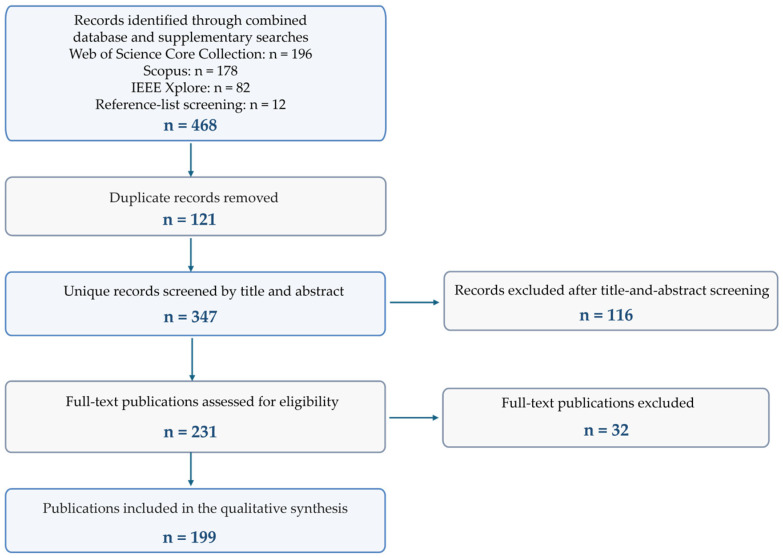
Literature-search and study-selection procedure used in this review.

Therefore, this review aims to systematically analyze how perception and localization errors propagate to reference paths, controller inputs, and vehicle closed-loop responses, and to compare the applicability, advantages, and limitations of typical path-tracking control methods, thereby addressing the lack of integrated research on error propagation and robust control for agricultural machinery in hilly orchards. The literature-selection procedure is summarized in Figure 2.

## 2. Key Observation Sources for Perception and Localization in Hilly Orchards and Their Error Effects

Reliable acquisition of perception and localization information is a prerequisite for autonomous navigation and path-tracking control of agricultural machinery in hilly orchards [42]. Path-tracking control depends not only on vehicle motion states, such as position, heading angle, velocity, and attitude, but also on environmental-structure information, including tree rows, inter-row corridors, obstacles, boundaries, and local terrain [43]. Compared with flat farmland or regularly structured orchards, factors such as canopy occlusion, terrain undulation, slope-induced slip, soft and slippery soil, limited inter-row space, and irregular tree-row structures in hilly orchards can degrade sensor observation quality and cause observation errors to propagate progressively to the path-tracking controller through vehicle pose estimation, environmental-structure perception, and reference path generation [44].

According to the observed objects, the observation sources for the perception and localization of agricultural machinery in hilly orchards can be divided into two categories: one is vehicle motion-state observation sources, mainly including GNSS, inertial measurement unit (IMU), and wheel odometry, which provide vehicle position, attitude, velocity, and odometry information; the other is environmental-structure perception sources, mainly including vision sensors, light detection and ranging (LiDAR), and depth perception sensors, which are used to extract environmental information such as tree-row structures, inter-row corridors, obstacles, boundaries, and local terrain [45].

### 2.1. GNSS, IMU, and Wheel Odometry

The GNSS, IMU, and wheel odometry are the most commonly used observation sources for motion-state estimation of agricultural vehicles [46,47,48]. The GNSS mainly provides global position constraints and can be used to estimate the vehicle position in the navigation coordinate system; IMU provides angular velocity, acceleration, and attitude-change information to maintain short-term motion continuity; and wheel odometry provides wheel angular velocity, vehicle speed, or odometry increments, serving as an important supplement for velocity feedback and trajectory dead reckoning. These three sources are usually fused through filtering or integrated navigation methods to provide fundamental state variables for path-tracking control, including vehicle position, heading angle, velocity, and attitude [49]. However, the reliability of these state variables depends on observation quality and the validity of the assumed vehicle–ground kinematic relationship.

Direct sensor-level error measurements obtained from explicitly sloped orchard field experiments remain limited. Therefore, the following results are used as representative quantitative evidence from orchard, under-canopy, mountainous agricultural, and sloped agricultural environments, with their original test contexts explicitly retained. In hilly orchards, these observation sources for vehicle motion-state estimation are susceptible to complex environmental influences. The GNSS is affected by canopy occlusion, branch and leaf shielding, terrain undulation, and multipath effects, which may lead to a decrease in the number of visible satellites, loss of real-time kinematic (RTK) fixed solutions, and positioning jumps. Kabir et al. reported that the moving-position root mean square error (RMSE) of a multi-GNSS receiver increased from 0.152 m in an open field to 0.182 m in an orchard and 1.130 m in a mountainous agricultural environment, showing the progressive deterioration of GNSS positioning under vegetation and terrain obstruction [50]. Bene et al. further reported that, under tree canopy, a low-cost dual-frequency RTK receiver exhibited average horizontal errors of approximately 0.17–0.18 m during the leaf-on period and approximately 0.07 m during the leaf-off period [42].

Although an IMU can provide high-frequency attitude and motion propagation information, its bias and noise accumulate through time integration, resulting in position, velocity, and heading drift; terrain undulation on slopes and vehicle body vibration further increase the uncertainty of attitude estimation. Zhang et al. reported that, when an orchard spraying robot traveled at 0.4 m/s, the position-error bound decreased from 5.29 cm using the BeiDou Navigation Satellite System (BDS) alone to 2.49 cm using BDS/IMU integration, while the heading-error bound decreased from 3° to 2° [51]. In a separate slope experiment, Igarashi et al. reported a heading-error root mean square (RMS) below 1.5° when roll and pitch angles were below 45°; during field tests on an approximately 25° agricultural slope, the cross-track-error RMS was 7.2 cm downhill and 6.6 cm uphill [52].

The main error source of wheel odometry arises from the failure of the wheel–ground kinematic constraint; under conditions of wet and slippery soil, soft ground, and slope traction, longitudinal slip and sideslip can cause inconsistency between wheel-odometry-derived displacement and the actual displacement [53,54]. Existing orchard and agricultural-vehicle studies have generally reported the downstream control consequences of slip rather than directly providing generalizable raw odometry-error ranges. Li et al. [55] combined cascaded model predictive control with slip-rate-based anti-slip drive control for an orchard mowing robot, maintaining the average lateral and longitudinal tracking errors within 0.05 m and 0.04 m, respectively. Liu et al. fused RTK–IMU pose measurements with track-speed feedback to estimate the left and right track slip ratios, and their slip-aware compensation method reduced the maximum lateral deviation by 78.1% and 63.1% at operating speeds of 0.35 and 0.75 m/s, respectively [56]. These values represent closed-loop performance under slip compensation rather than raw wheel-odometry measurement errors.

These observation errors first affect the results of vehicle pose estimation and are then further propagated to the inputs of the path-tracking controller. GNSS position bias alters the estimated relative position between the vehicle and the reference path and is therefore transformed primarily into lateral-error bias. Abrupt GNSS position jumps may produce discontinuities in the calculated lateral error, leading to steering-command spikes or transient oscillations. IMU heading drift and high-frequency attitude noise affect heading estimation, coordinate transformation, and path-direction judgment, thereby causing heading-error bias or oscillatory steering commands. Wheel-odometry errors may affect vehicle speed, odometry propagation, and short-term motion prediction, thereby influencing reference speed execution, look-ahead distance setting, and model prediction accuracy. Especially under slope-induced slip conditions, the wheel-odometry output may no longer represent the actual motion state of the vehicle; without slip identification or confidence adjustment, such errors may further affect the estimation of vehicle position, velocity, and heading. Therefore, vehicle state estimation in hilly orchards should not rely on a single observation source but should improve the reliability of pose estimation through the complementary fusion of GNSS, IMU, wheel odometry, and environmental-structure perception information.

### 2.2. Vision Sensors

Vision sensors mainly include monocular cameras, binocular cameras, and multi-camera systems, and can acquire color, texture, edge, and semantic information from orchard environments. In orchard agricultural machinery navigation, vision sensors are commonly used for trunk recognition, tree-row detection, ground–vegetation segmentation, inter-row corridor extraction, obstacle recognition, and target detection for field operations [57,58]. Unlike the GNSS, which mainly provides the global position of the vehicle, vision sensors are more suitable for extracting local environmental structural information, and their outputs can be further used to fit tree-row centerlines, determine traversable areas, construct local reference paths, and calculate the tangential direction of the path [59,60,61]. From the perspective of path-tracking control, the most representative vision-related error variables include trunk or reference-point localization error, navigation-line lateral error, tree-row or path-direction error, frame-to-frame path jitter, and visual-processing latency. Detection and segmentation metrics, such as precision, recall, mean average precision (mAP), and mean intersection over union (mIoU), characterize front-end perception quality, whereas navigation-line position and direction errors are more directly related to the inputs of the path-tracking controller.

The main limitations of vision sensors in hilly orchards are their sensitivity to illumination, occlusion, and texture variations [62,63]. Direct quantitative measurements of vision-related errors from explicitly sloped orchard field experiments remain limited. Therefore, the following results are used as representative evidence from regular orchards and transferable agricultural environments, with their original test contexts explicitly retained. Canopy projections, mottled shadows, backlighting, strong reflections, seasonal changes in branches and leaves, weed coverage, and wet and slippery ground can all alter image features, resulting in missed trunk detection, boundary misclassification, or unstable segmentation of corridor areas. Especially in unstructured inter-row environments, shadows may be misclassified as boundaries, weeds may be misidentified as tree rows or obstacles, and branch and leaf occlusion may lead to discontinuous trunk detection [64]. Yang et al. reported segmentation accuracies of 96%, 92%, and 92% under low, normal, and strong illumination, respectively, while the extracted navigation path exhibited an average pixel error of 9.5 pixels and an average distance error of 5.03 cm [65]. Zhou et al. reported a mean tree-row fitting accuracy of 91.67%; the mean centerline pixel errors under weak, normal, and strong illumination were 5.2, 3.4, and 2.1 pixels, respectively [66]. Using a U-Net-based method, Xu et al. achieved precision, recall, and mIoU values of 91.71%, 90.23%, and 84.84%, respectively; however, the extracted navigation line still showed an average distance error of 56 mm [67]. In complex and variable agricultural scenarios, Lu et al. achieved an mIoU of 97.55% at an inference speed of 32.60 frames per second (FPS), whereas the mean absolute yaw-angle error of the extracted navigation line remained 2.06° [68]. These results demonstrate that high pixel-level detection or segmentation accuracy does not necessarily eliminate geometric errors in the position and direction of the generated navigation line.

These vision-derived errors first affect environmental-structure perception and are subsequently propagated through reference path construction to the path-tracking controller. When lateral deviations occur in trunk detection or tree-row boundary recognition, the generated inter-row centerline may be shifted as a whole, producing a bias in the reference path position and subsequently in the lateral error calculated by the controller; when tree-row direction estimation is unstable, the tangential direction of the reference path may fluctuate, affecting subsequent heading-error construction. Gu et al. converted binocular trunk observations into ground-plane trunk coordinates and subsequently fitted the inter-row navigation line, obtaining average estimation errors of 0.02 m for lateral deviation and 0.57° for the heading angle [69]. When visual segmentation results are discontinuous between adjacent frames, local jumps or abrupt curvature changes may appear in the reference path, weakening the stability of path-tracking controller inputs. At the closed-loop level, Xie et al. reported average lateral and heading errors of 0.059 m and 0.2787 rad, respectively, when an orchard robot traveled at 0.5 m/s during the entire convergence process. After the robot entered a steady state, the corresponding average errors decreased to 0.0102 m and 0.0253 rad, respectively [70]. These values represent the integrated closed-loop performance of visual fruit-tree recognition, navigation-line generation, and vehicle navigation rather than raw camera measurement errors. Therefore, visual outputs should undergo illumination adaptation, geometric-consistency checking, temporal filtering, and confidence evaluation before being used for reference path generation. When visual confidence decreases or the extracted navigation line becomes discontinuous, the system should temporarily retain the last reliable path, reduce the reference speed, or switch to a complementary perception source.

### 2.3. LiDAR Sensors

LiDAR describes the spatial structure of orchard environments by acquiring environmental point-cloud information, and can extract trunks, support structures, ground surfaces, obstacles, and inter-row geometric boundaries with relatively high stability. Compared with vision sensors, LiDAR is insensitive to illumination variations and has advantages in trunk localization, inter-row boundary recognition, obstacle detection, local map construction, and traversable-area determination [71]. Two-dimensional LiDAR has a simple structure and low computational cost, making it suitable for low-speed inter-row navigation, whereas three-dimensional LiDAR can provide richer spatial structural information and is suitable for mapping and terrain representation in complex orchard environments [72,73,74,75]. From the perspective of path-tracking control, representative LiDAR-related error variables include ranging error, point-cloud registration translation and rotation errors, trunk or boundary localization error, navigation-centerline error, terrain-slope estimation error, and point-cloud processing latency. Among these variables, registration and geometric-feature errors are more directly related to vehicle-state feedback and reference path construction than generic point-cloud density or detection-rate metrics.

Nevertheless, the reliability of these intermediate results remains sensitive to terrain-induced attitude changes, occlusion, point-cloud sparsity, and scan blind spots. Direct quantitative measurements of LiDAR errors obtained from explicitly sloped orchard field experiments remain limited. Therefore, the following results are used as representative evidence from regular orchards and transferable row-crop environments, with the original test contexts and metric levels explicitly retained. Vehicle pitch and roll change the relative relationship between the laser scanning plane and the ground, causing the coordinates of the same trunk or boundary to shift across point-cloud frames; terrain undulation affects ground-point segmentation and obstacle recognition; and branch and leaf occlusion, moving branches, sparse point clouds, and scan blind spots can lead to missing trunk point clouds or discontinuous boundaries. In a transferable row-crop study, Yang et al. used three-dimensional LiDAR to detect crop-row centerlines at four maize growth stages and reported an average correct detection rate above 86% and an average processing time below 120 ms [76]. Although these results were obtained in maize fields rather than orchards, they show that point-cloud occlusion and crop-density variation affect both the availability and real-time extraction of row-structure features. In addition, repetitive tree-row structures may induce scan-matching degeneracy, resulting in drift in LiDAR/IMU localization or local mapping [77,78]. Sun et al. developed a tightly coupled GNSS/LiDAR/IMU odometry framework for orchard environments and reported an absolute translation error of 0.068 m and an absolute rotation error of 0.856° [79]. These values describe the output error of the integrated odometry system rather than the raw ranging error of LiDAR, but they quantify the residual uncertainties associated with point-cloud registration and multi-sensor fusion in the estimated vehicle pose.

These LiDAR-related errors first affect vehicle pose estimation and environmental-structure representation and are subsequently propagated through local mapping and reference path construction to the path-tracking controller. When trunk point clouds are missing or boundary extraction fluctuates, lateral offsets may occur in centerline construction, producing a bias in the reference path position and subsequently in the lateral error calculated by the path-tracking controller. Chen et al. constructed orchard navigation points from trunk positions retained in a LiDAR-derived grid map. Using a 32-line LiDAR and a 3 m navigation-point interval, the system achieved an average absolute lateral deviation of 1.83 cm, a standard deviation of 1.60 cm, and a maximum deviation of 10.30 cm [80]. These values represent the closed-loop outcome of LiDAR mapping, trunk-position extraction, centerline construction, and vehicle navigation rather than the raw error of trunk detection alone. When point-cloud registration or local maps drift, deviations may occur in the relative relationship between the vehicle and the environmental map, thereby introducing biases into the lateral-error and heading-error inputs used by the controller. Terrain-induced attitude variations, surface unevenness, and changes in ground conditions can affect both the geometric consistency of LiDAR-derived environmental guidance and the vehicle response to that guidance. At the system level, Jiang et al. used LiDAR-based trunk detection for autonomous orchard navigation and reported position RMSE values of 12.0 and 11.6 cm for right and left turns on concrete, 12.6 and 15.5 cm on grass, and 13.8 and 11.4 cm in an artificial tree orchard, respectively [81]. These differences suggest that the final navigation error is jointly determined by the quality of LiDAR-derived environmental guidance and the vehicle–ground motion response, rather than by LiDAR perception alone. Therefore, in hilly orchards, LiDAR should not only provide geometric structural information but also be combined with IMU-based attitude compensation, local map updating, and abnormal point-cloud removal to ensure the continuity of the reference path and local environmental constraints.

### 2.4. Depth Perception Sensors

Depth perception sensors mainly include red–green–blue-depth (RGB-D) cameras, structured-light cameras, time-of-flight cameras, and binocular depth cameras, all of which can simultaneously provide image semantic information and distance information [82]. Their advantage lies in the ability to combine two-dimensional image recognition results with depth information to directly estimate the positions of trunks, branches, obstacles, and corridor boundaries relative to the vehicle, making them suitable for short-range obstacle avoidance, local traversable-area determination, and local reference path correction in low-speed orchard robots [83,84]. From the perspective of path-tracking control, representative depth-related error variables include depth or ranging error, effective outdoor sensing range, valid-depth filling rate, trunk or obstacle localization error, edge-depth discontinuity, and depth-processing latency. Among these variables, obstacle-position error, boundary-position error, and valid-depth availability are more directly related to local path correction and safety-constraint construction than generic image-level detection metrics.

Direct quantitative evaluations of depth sensors in explicitly sloped orchard navigation experiments remain scarce. Therefore, the following results are used as representative evidence from regular orchards and transferable outdoor agricultural environments, with their original test contexts and metric levels explicitly retained. Outdoor hilly orchards nevertheless present unfavorable conditions for depth perception sensors. Strong illumination, low-texture regions, leaf reflections, edge distortion in the field of view, limited effective ranging distance, and vehicle body vibration can all reduce the accuracy of depth estimation. Vit et al. compared several RGB-D sensors under outdoor agricultural conditions and showed that depth-data quality depended strongly on both working distance and illumination, with the Intel RealSense D435 (Intel Corporation, Santa Clara, CA, USA) providing comparatively reliable performance for close-range field measurements [45]. Fan et al. further reported that the effective ranging interval of the RealSense D435i (Intel Corporation, Santa Clara, CA, USA) under field conditions was approximately 0.160–1.400 m, with an in-field depth filling rate of approximately 90%. The Kinect V2 (Microsoft Corporation, Redmond, WA, USA) exhibited relatively high ranging accuracy over approximately 0.497–1.200 m, but its field depth filling rate was below 24.9% [85]. These results indicate that the usability of a depth sensor depends not only on the error of valid measurements but also on whether sufficient valid depth pixels remain available under outdoor illumination. When holes, distance jumps, or edge distortions occur in depth maps, the estimation of nearby obstacle positions, local traversable boundaries, and safety distances may become biased, thereby affecting local path correction and obstacle-avoidance control [86]. In an orchard-specific study, Huang et al. combined deep-learning-based trunk detection with depth estimation and reported an overall trunk-detection mAP of 81.6%, an inference time of 60 ms, and a trunk-location error of 9 mm at a distance of 2.8 m [82]. The 9 mm value represents the integrated spatial localization error produced by trunk detection and depth estimation rather than the raw ranging error of the depth sensor alone.

These depth-related errors primarily affect short-range environmental representation and are subsequently propagated through obstacle-boundary construction, traversable-corridor estimation, and local path correction to the path-tracking and obstacle-avoidance modules, rather than directly providing a global reference for path tracking. If depth estimation misclassifies traversable areas as obstacles, the reference path may produce unnecessary detours or lateral offsets; if low obstacles or branches are not detected, the vehicle may follow the theoretical reference path while still facing a risk of collision. In a transferable under-canopy agricultural study, Gai et al. used a time-of-flight depth camera for crop-row detection and mapping. The resulting crop-row maps exhibited mean absolute errors of 3.4 cm in corn and 3.6 cm in sorghum, while the corresponding lateral-position estimates showed mean absolute errors of 5.0 and 4.2 cm, respectively [87]. These results illustrate how depth-derived row-structure errors can be further transformed through mapping into errors in the lateral position information used for navigation. Therefore, depth observations should undergo invalid-depth filtering, edge-consistency checking, temporal smoothing, confidence evaluation, and cross-validation with vision or LiDAR before being used for obstacle-boundary construction and local path correction. When the valid-depth filling rate or ranging confidence decreases, the system should enlarge the obstacle safety margin, reduce the reference speed, or switch to a complementary sensing source rather than directly using incomplete depth maps. These direct depth-related errors are further transformed through obstacle representation, traversable-corridor construction, safety-boundary setting, and local path generation.

### 2.5. Summary

Table 1 summarizes the primary navigation measurements or outputs, representative reported performance metrics, major error sources, and supporting references for the principal observation sources used in hilly orchard navigation. The operating characteristics of these observation sources differ substantially. The GNSS provides global positioning coverage, but effective RTK positioning depends on satellite visibility, continuity of the differential-correction link, and maintenance of a fixed solution. The IMU does not have a fixed spatial sensing range and is mainly used for high-frequency, short-term state propagation between external positioning updates; however, its standalone position, velocity, and heading errors accumulate through time integration. Wheel odometry similarly provides continuous local dead reckoning rather than a fixed sensing range, and its reliability deteriorates with traveled distance, wheel or track slip, soil deformation, and changes in ground adhesion. Vision and LiDAR provide local environmental information at device- and task-dependent spatial scales, whereas depth sensors are mainly suitable for short-range obstacle, trunk, and corridor perception, with their effective ranges strongly affected by illumination, target reflectivity, sensor configuration, and outdoor environmental conditions.

The quantitative values summarized in Table 1 should not be interpreted as directly comparable raw sensor-measurement accuracies. Studies that directly report raw sensor-measurement errors in explicitly quantified hilly orchard environments remain scarce. Existing studies more frequently report navigation-line extraction errors, trunk- or boundary-localization errors, point-cloud registration errors, integrated-localization errors, or final navigation-system errors. Moreover, different sensing modalities and processing stages use different metrics. Consequently, only a limited proportion of the available quantitative evidence can be classified as raw sensor-measurement error, and receiver-level measurements, perception-algorithm outputs, integrated-localization results, and closed-loop navigation errors should be explicitly distinguished.

## 3. Transformation of Perception and Localization Outputs into Controller Inputs

For agricultural machinery operating in hilly orchards, front-end perception and localization results cannot be directly used for closed-loop control; instead, they must undergo processes such as multi-source fusion localization, tree-row and corridor structure recognition, local environment representation, reference centerline fitting, path smoothing, and reference speed planning before stable and continuous tracking targets that satisfy vehicle motion constraints can be generated [88].

Therefore, from the perspective of path-tracking control requirements, this section focuses on analyzing how vehicle pose estimation methods provide controllers with state information such as position, heading, velocity, and attitude; how orchard environmental-structure perception and representation methods provide environmental information such as tree rows, corridors, obstacles, boundaries, and local terrain; and how reference path generation methods further generate reference centerlines, path directions, reference curvature, reference speed, and safety constraints. The accuracy and continuity of the above information directly affect error construction, control-input smoothness, and closed-loop robustness in subsequent path-tracking control [89,90]. Accordingly, this section does not re-evaluate the sensor-level accuracy discussed in Section 2. Instead, it examines how direct observation and perception outputs are transformed into four categories of controller-related information: vehicle-state variables, environmental geometric variables, reference path variables, and safety constraints. Each transformation process is discussed in terms of its input information, structured output, residual or newly introduced errors, and downstream effects on path-tracking control.

### 3.1. Vehicle-State Estimation and Multi-Source Fusion Localization Methods

Vehicle pose estimation is a key step in transforming multi-source observation data into the vehicle states required for path-tracking control [91,92]. From the perspective of path-tracking control, the core outputs of vehicle-state estimation include vehicle position, heading, velocity, roll, and pitch. Where available, the outputs may also include indicators of solution reliability, such as localization confidence, covariance, GNSS solution status, sensor-health flags, or slip-detection results. In flat farmland, path-tracking control usually relies mainly on planar position, heading angle, and velocity; however, in hilly orchards, longitudinal slope, cross-slope inclination, and surface undulation affect sensor coordinate transformation, point-cloud registration, visual feature matching, and vehicle motion response, and therefore the stability of attitude estimation also influences path-tracking control accuracy [93,94,95].

In orchard navigation research, multi-source fusion localization has become an important technical approach for improving the robustness of vehicle pose estimation. GNSS/IMU fusion can integrate global position constraints with short-term motion propagation capabilities, and is commonly used to alleviate localization discontinuities caused by canopy occlusion and short-term GNSS signal degradation. To mitigate localization degradation in the fusion between a GNSS and an inertial navigation system (INS) under orchard conditions, Gao et al. [96] introduced a gated recurrent unit (GRU)–Transformer hybrid model to improve the localization accuracy of GNSS/INS integrated navigation in orchard environments, indicating that data-driven methods can complement error compensation in traditional integrated navigation under complex agricultural scenarios.

**Figure 3:**
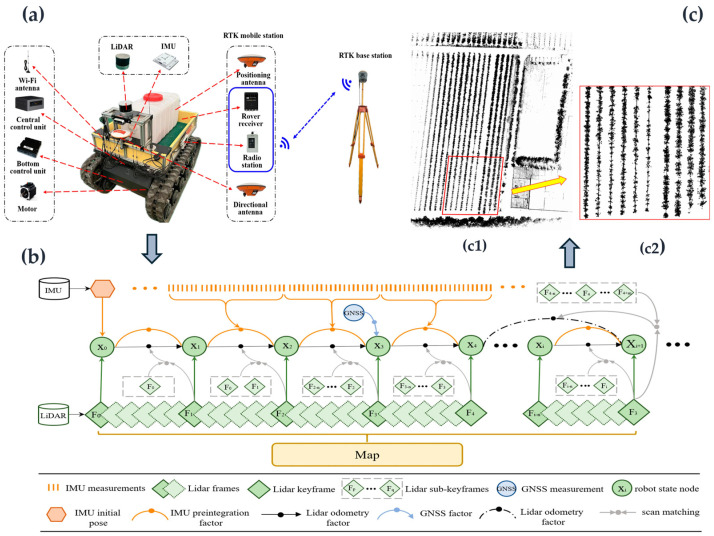
Workflow of orchard robot pose estimation and map construction based on LiDAR/IMU/GNSS fusion: (**a**) multi-source sensor configuration; (**b**) LiDAR/IMU/GNSS fusion localization framework; (**c**) orchard map construction results, where (**c1**) shows the global orchard grid map and (**c2**) shows an enlarged view of the local tree-row structure. (Reprinted from Ref. [97] under the Creative Commons Attribution 4.0 International license (CC BY 4.0)).

Beyond satellite–inertial integration, LiDAR, vision, and local geometric constraints have been incorporated into orchard localization frameworks to maintain pose continuity under GNSS degradation. Wang et al. [97] constructed a fusion navigation system based on LiDAR/IMU/GNSS for an orchard spraying robot, used Tightly Coupled LiDAR Inertial Odometry via Smoothing and Mapping (LIO-SAM) for orchard map construction, and combined RTK-GNSS with Kalman filtering to obtain the initial coordinates and heading of the robot, thereby providing reliable state inputs for subsequent path planning and autonomous navigation. Its typical process is shown in Figure 3.

In orchard scenarios with constrained GNSS signals, repetitive tree-row structures, or salient local geometric features, LiDAR/IMU fusion localization and LiDAR-based simultaneous localization and mapping (SLAM) have also been used to enhance local pose estimation capabilities. LiDAR can use geometric structures such as trunks, ground surfaces, boundaries, and inter-row corridors for environmental matching, whereas IMU provides prior information for attitude constraints, point-cloud deskewing, and short-term motion prediction. Choi et al. [98] applied LiDAR/IMU sensor fusion to SLAM in orchard autonomous navigation, improving the stability of local maps and vehicle pose estimation. In addition to geometric constraints, visual semantic information has also been introduced into multimodal fusion frameworks. Wang et al. [99] combined visual–inertial odometry (VIO) with model predictive control (MPC) and verified autonomous headland turning in a GNSS-denied orchard environment, demonstrating the direct coupling between perception and localization results and the state inputs of path-tracking control.

Overall, the principal function of multi-source localization in the reviewed navigation chain is to convert GNSS, IMU, wheel-odometry, LiDAR, and visual observations into a continuous vehicle-state estimate. However, fusion does not eliminate upstream uncertainty. Position bias remains in the estimated relative position between the vehicle and the reference path and therefore affects lateral-error calculation; heading bias affects heading-error construction and coordinate transformation; and velocity error influences look-ahead-distance adjustment, reference-speed execution, and model-based motion prediction. Where available, reliability indicators can accompany the estimated vehicle state and support downstream monitoring or conservative adjustment when localization quality deteriorates.

### 3.2. Orchard Environmental-Structure Perception and Representation Methods

The role of orchard environmental-structure perception and representation is to extract environmental information such as vehicle driving direction, traversable areas, and local terrain from vision, LiDAR, depth perception, or multi-source fusion data [100]. For hilly orchards, environmental-structure perception should not focus solely on object detection; more importantly, perception results should be transformed into structured navigation information that can be used for path generation and path-tracking control, thereby providing stable reference centerlines, path directions, local traversable spaces, and safety-constraint information for path-tracking control [101].

Based on the direct visual, LiDAR, and depth observations reviewed in Section 2.2, Section 2.3 and Section 2.4, the environmental-structure representation further converts discrete detections, segmentation masks, point clouds, and depth measurements into structured geometric variables. These variables include trunk coordinates, tree-row directions, corridor boundaries, obstacle positions, terrain characteristics, and safety margins, which can subsequently be used for reference path construction.

#### 3.2.1. Tree-Row and Trunk Structure Recognition Methods

Tree-row and trunk structure recognition is one of the most typical environmental-structure perception tasks in orchard inter-row navigation, and its main role is to extract structured information, such as trunk positions, tree-row directions, and inter-row corridors, from complex vegetation backgrounds, thereby providing an environmental basis for navigation-line generation and vehicle driving-direction judgment [102,103]. Traditional methods often use color thresholding, edge detection, Hough transform, least-squares fitting, random sample consensus (RANSAC), or point-cloud clustering to extract trunk and tree-row structures, whereas deep learning methods identify trunks, canopies, ground surfaces, and inter-row regions through object detection, semantic segmentation, or instance segmentation [76,104].

Existing studies have mostly adopted vision-based methods to recognize tree-row structures and have applied them to inter-row navigation in regular or semi-regular orchards. To address navigation path extraction in complex and variable agricultural scenarios, Lu et al. [68] combined Attention-Feature-Enhanced U-Net (AFU-Net) with key contour-point constraints to extract navigation paths, providing a vision-based perception method for traversable-area recognition in complex vegetation backgrounds. For navigation path extraction in complex orchard environments, Lv et al. [105] developed a method combining object detection with a dual-shortest-distance intersection algorithm to improve the accuracy and adaptability of inter-row navigation path recognition in orchards.

**Figure 4:**
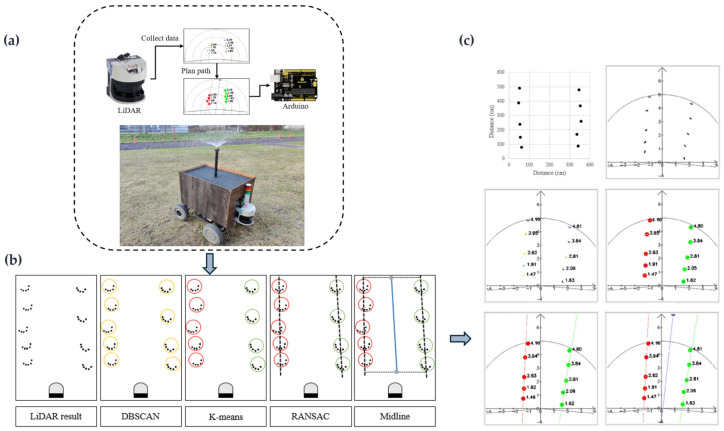
Workflow of orchard environmental-structure representation and navigation path extraction based on LiDAR trunk detection: (**a**) LiDAR-based orchard environmental-structure perception platform; (**b**) workflow of trunk detection, left and right tree-row grouping, boundary-line fitting, and inter-row centerline extraction; (**c**) navigation path extraction results. In panels (**b**,**c**), the black dots represent LiDAR-detected trunk points; the yellow circles indicate the DBSCAN clustering results; the red and green circles or points denote the two tree-row groups; the fitted lines represent the corresponding tree-row boundaries; and the blue line denotes the extracted inter-row midline or navigation path. (Reprinted from Ref. [81] under the Creative Commons Attribution 4.0 International license (CC BY 4.0)).

For orchards with distinct trunk structures, LiDAR point-cloud methods have also been widely used for trunk detection and tree-row structure extraction. Bargoti et al. [106] proposed a trunk detection pipeline for trellis-structured apple orchards, providing a methodological reference for trunk-structure-based inter-row perception in regular orchards. Therefore, in orchard scenarios with clear trunk structures and relatively stable inter-row geometric features, LiDAR point-cloud-based trunk clustering, left–right tree-row matching, and centerline fitting can provide structured environmental information for local path generation and autonomous navigation of orchard robots. At this stage, the relevant outputs are trunk coordinates, left–right tree-row associations, tree-row directions, and candidate inter-row center points, rather than the final reference path tracked by the controller. These structured outputs generally require coordinate transformation, geometric association, abnormal-point rejection, and path fitting before they can be converted into controller reference information. Jiang et al. [81] applied LiDAR-based trunk detection to the autonomous navigation of an orchard spraying robot, and their study clearly illustrates the typical process by which orchard environmental-structure perception results are transformed into navigation path information: LiDAR is first used to acquire spatial point sets of trunks and surrounding obstacles, then clustering and fitting methods are employed to identify the left and right tree-row boundaries, and finally the inter-row centerline is calculated and used as the robot navigation path. To intuitively illustrate the process of environmental representation and navigation path extraction based on trunk geometric structures, Figure 4 summarizes the LiDAR perception platform, trunk detection procedure, and path extraction results.

Overall, in hilly orchards, trunk occlusion, overlapping branches and leaves, differences in tree age, missing or replanted trees, curved tree rows, and missing point clouds can all lead to errors in trunk position estimation or tree-row direction estimation. When lateral offsets occur in trunk detection results, the inter-row centerline may deviate as a whole from the true center of the corridor, causing systematic errors in the calculation of the vehicle’s lateral deviation; when tree-row direction estimation is unstable, the tangential direction of the reference path may fluctuate, thereby affecting vehicle heading correction. When candidate centerline points are discontinuous or local fitting is unstable or excessively smoothed, local bends or abrupt curvature changes may appear in the reference path, potentially causing discontinuities in controller inputs. Therefore, tree-row recognition methods should not only improve detection accuracy, but also preserve the geometric consistency and temporal continuity of trunk and tree-row outputs and retain confidence estimates where available. The subsequent transformation of these outputs into reference path position and direction is discussed in Section 3.3.1.

#### 3.2.2. Inter-Row Corridor and Traversable-Area Recognition Methods

The goal of inter-row corridor and traversable-area recognition is to extract regions within complex orchard environments in which vehicles can travel safely, and its outputs usually include corridor boundaries, traversable-area masks, safety corridors, local occupancy grids, or traversability cost maps [107,108]. Compared with methods that rely solely on tree-row centerline fitting, traversable-area recognition places greater emphasis on the integrated constraints imposed on the driving space by vehicle dimensions, implement working width, safety distance, and local obstacles [109,110]. Therefore, such methods not only serve navigation-line extraction but also provide an environmental basis for local path planning, obstacle-avoidance decision-making, and safety-constraint setting in path-tracking control [111].

In terms of visual perception, image-based traversable-area recognition usually distinguishes categories such as ground surfaces, vegetation, trunks, canopies, and obstacles based on color, texture, boundary, or semantic information, and further derives the drivable area for vehicles. Xu et al. [112] constructed a vision-based navigation framework for autonomous tractor navigation in peach orchards, indicating that visual perception results can provide environmental structural information for the inter-row navigation of orchard vehicles. To address navigation path extraction on hardened orchard roads, Yang et al. [65] combined a scanning method with a neural network to identify road-surface regions, providing visual support for navigation path generation in orchard roads or relatively regular corridor environments. Furthermore, Guan et al. [113] proposed a lightweight semantic segmentation model for automatic navigation in pomelo orchards to segment key orchard elements, demonstrating that semantic segmentation methods can enhance traversable-space recognition in environments where canopies, ground surfaces, and inter-row areas are intermingled.

In addition to vision-based methods, LiDAR-derived semantic cost maps have also been used to represent traversable spaces in orchards. Nishiwaki et al. [114] used unmanned aerial vehicle LiDAR data to construct a semantic cost map and applied it to orchard navigation of an unmanned ground vehicle, showing that cost maps integrating spatial geometry and semantic information can provide richer environmental constraints for path planning and safe passage of orchard vehicles. Regardless of the sensing modality, traversable-area observations must be converted into controller-related geometric constraints, including the left and right corridor boundaries, available corridor width, vehicle-clearance margin, obstacle-inflation regions, and local traversability confidence. Collectively, the reviewed studies indicate that vision masks can provide semantic-boundary information, LiDAR can provide geometric-clearance information, and depth observations can supplement the representation of nearby obstacles and narrow passages [82,87,112,113,114]. These outputs jointly define the feasible corridor within which a local reference path can be generated.

Accordingly, in hilly orchards, the local path is not necessarily equivalent to the geometric centerline between two rows of fruit trees. Drooping canopies, intruding branches, protruding support structures, stones, ditches, ridges, tree basins, weeds, and local waterlogging may all render the geometric centerline no longer the safest path. Therefore, an overly conservative boundary may cause unnecessary path offsets and frequent vehicle corrections, whereas an overly optimistic boundary may reduce obstacle clearance and increase collision or stability risks. Accordingly, traversable-area representation errors first alter the feasible corridor and safety margin and are subsequently converted into local-path offsets, unnecessary detours, or insufficient safety constraints, as discussed in Section 3.3.2.

#### 3.2.3. Obstacle, Boundary, and Three-Dimensional Terrain Representation Methods

Representations of obstacles, boundaries, and three-dimensional terrain describe information around the vehicle, such as trunks, branches, support structures, stones, ditches and ridges, tree basins, slopes, and headland boundaries. Common representation forms include point-cloud maps, semantic point clouds, occupancy grids, cost maps, local elevation maps, and traversability maps. For path-tracking control, such structural information is used not only for obstacle avoidance, but also for determining the feasible region of the reference path, speed limits, vehicle attitude risks, and steering-space constraints [115]. Therefore, local environmental-structure representation serves as an important intermediate layer linking orchard environmental-structure perception, local path generation, and robust path-tracking control [116,117,118].

**Figure 5:**
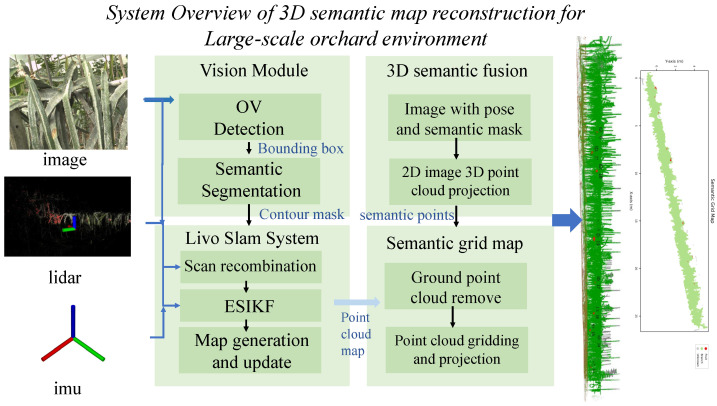
Framework for three-dimensional semantic map reconstruction in a large-scale orchard environment. Image, LiDAR, and IMU observations are fused to generate three-dimensional semantic point-cloud and semantic grid-map representations for orchard environmental understanding and autonomous navigation. (Reproduced from Ref. [119] under the Creative Commons Attribution 4.0 International license (CC BY 4.0)).

In research on orchard autonomous navigation, local map construction and obstacle representation are often integrated with localization, path planning, and control decision-making. Wang et al. [119] investigated three-dimensional semantic map reconstruction for orchard environments by fusing image, LiDAR, and IMU observations. Their framework combines visual object detection and semantic segmentation with LiDAR–inertial SLAM, and further projects semantic image information onto the three-dimensional point cloud to generate semantic point-cloud and grid-map representations. The overall workflow is illustrated in Figure 5. The framework illustrates how heterogeneous sensor observations can be transformed into semantic point-cloud and grid-map representations that are directly usable by downstream localization, path-planning, and navigation modules.

Building on this type of structured environmental representation, Pan et al. [120] proposed a perception and semantic mapping method for the autonomous operation of orchard robots, demonstrating that semantic maps can transform raw sensor observations into structured environmental information more suitable for robot navigation, path planning, and task execution. These studies indicate that the value of semantic local maps lies not only in improving environmental perception accuracy but also in transforming obstacles, boundaries, and task-relevant objects in orchards into spatial constraints that can be invoked by path-planning and control systems.

In local path planning and obstacle-avoidance applications, point-cloud maps, occupancy maps, and cost maps can further describe the distribution of obstacles around the vehicle and their associated traversability risks. Jiang et al. [121] applied three-dimensional LiDAR-based SLAM and Normal Distributions Transform–Iterative Closest Point (NDT-ICP) point-cloud registration to the navigation system of an orchard spraying robot, demonstrating the role of three-dimensional point-cloud mapping in vehicle localization, environmental modeling, and navigation path generation. Liang et al. [122] integrated map construction and localization methods with autonomous obstacle avoidance and path planning for orchard mobile robots, indicating that local environment representation can directly affect the robot’s judgment of obstacles, boundaries, and drivable space. Such studies suggest that orchard robot navigation should not rely solely on a single geometric centerline of tree rows, but should dynamically adjust the reference path and obstacle-avoidance strategy by incorporating local maps and obstacle distributions.

For hilly orchards, representing only two-dimensional obstacle positions is still insufficient to meet the requirements of safe navigation in complex terrain; information such as terrain elevation, slope, roughness, and traversability should also be incorporated into local environmental-structure representation. Cao et al. [123] applied traversability analysis, an improved Lightweight and Ground-Optimized LiDAR Odometry and Mapping (LeGO-LOAM) method, and the rapidly exploring random tree (RRT) algorithm to environmental mapping and path planning for orchard robots, indicating that terrain traversability evaluation can provide richer constraint information for path generation in complex orchard environments. For controller-oriented representation, three-dimensional terrain information should be organized into longitudinal slope, cross-slope inclination, local elevation variation, surface roughness, terrain discontinuities, and traversability or stability levels. These quantities should not be treated merely as mapping outputs. They can be further converted into obstacle boundaries, permissible speed ranges, lateral-stability margins, non-traversable regions, or other vehicle-specific constraints.

Compared with flat farmland, cross slopes, terrain undulations, and uneven ground in hilly orchards can alter vehicle attitude response and wheel–ground contact conditions; therefore, local environment representation needs to be further extended from obstacle detection to the representation of terrain risks and vehicle stability [124].

Overall, local environmental-structure errors mainly affect path-tracking control through two pathways. First, errors in obstacle and boundary recognition can change the feasible region of the local reference path, causing lateral offsets or local detours in the generated path, thereby affecting the positional relationship between the vehicle and the reference path. Second, terrain-representation errors can affect the estimation of slope, cross-slope inclination, roughness, and traversability, resulting in unreasonable settings of reference speed and control constraints. For example, underestimation of cross-slope risk may result in insufficient speed or stability constraints, whereas excessive smoothing of terrain undulations may attenuate or remove local terrain features from the environmental representation. Therefore, local environment representation in hilly orchards should be extended from two-dimensional obstacle representation to the joint representation of three-dimensional terrain, vehicle stability, and control constraints.

### 3.3. Reference Path and Controller-Input Construction

Vehicle pose estimation and environmental-structure representation provide state and environmental information for path tracking, but controllers cannot directly track trunk points, semantic masks, or point-cloud maps. Path-tracking control requires reference paths that are continuous, smooth, geometrically well-defined, and compliant with vehicle motion constraints. Therefore, reference path generation serves as a key bridge connecting perception and localization with path-tracking control. Its task is to transform tree-row structures, traversable areas, obstacle boundaries, and local terrain constraints into reference paths and associated speed profiles, and further provide information such as path direction, curvature variation, reference speed, and safety constraints [125,126]. A locally extracted visual or LiDAR navigation line should therefore not be regarded as equivalent to the final controller reference path. The latter may additionally involve coordinate transformation, multi-frame updating, obstacle avoidance, curve fitting, smoothing, curvature limitation, speed assignment, and vehicle-feasibility checking.

#### 3.3.1. Tree-Row Centerline Fitting and Reference Path Generation Methods

Tree-row centerline fitting is one of the most common methods for inter-row path generation in orchards, and its core idea is to construct a local reference path representing the desired driving direction of the vehicle based on structured perception results such as trunk positions, tree-row boundaries, canopy edges, or inter-row center points [127,128]. Based on the geometric structure of fruit-tree rows, local reference centerlines can be generated through line fitting, polynomial fitting, RANSAC, B-splines, Bézier curves, or multi-frame centerline updating. In regular orchards, vehicles usually travel along the center between the left and right tree rows; in irregular or hilly orchards, the centerline needs to be dynamically corrected according to tree-row curvature, missing trees, ground undulation, and variations in inter-row width.

Existing studies have mostly used the tree-row centerline as the basic reference path for inter-row navigation of orchard robots. Chen et al. [80] investigated a multi-point autonomous navigation method for orchard row centerlines based on LiDAR. Their method converted three-dimensional orchard point clouds into a two-dimensional grid map, extracted trunk positions, and generated multiple row-centerline navigation points, demonstrating that tree-row geometric structures can provide direct navigation references for orchard vehicles.

Barawid et al. [129] used a two-dimensional laser scanner to construct an orchard autonomous navigation system, demonstrating the feasibility of generating navigation paths based on the geometric features of trunks or inter-row boundaries. Such LiDAR- or laser-scanning-based centerline extraction methods are insensitive to illumination variations and can obtain the spatial distribution of trunks or inter-row boundaries reliably; therefore, they are suitable for orchard environments with clear trunk structures and distinct inter-row geometric features.

In vision- and learning-based methods, centerline or navigation path extraction usually relies on information such as canopy boundaries, ground regions, trunk positions, and inter-row vanishing directions in images. Liu et al. [130] proposed a single-stage navigation path extraction network for orchard agricultural robots, showing that deep learning methods can directly extract navigation paths from visual information under complex backgrounds. Xie et al. [70] investigated fruit-tree recognition and navigation for agricultural robots in complex environments, indicating that fruit-tree recognition results can be further used for inter-row navigation direction judgment and local path generation. Compared with traditional geometric fitting methods, vision- and learning-based methods have stronger feature representation capabilities in complex vegetation backgrounds; however, their outputs may still be affected by illumination variations, branch and leaf occlusion, canopy overlap, and seasonal background differences. Therefore, path continuity constraints or multi-frame updating mechanisms should be incorporated to improve the stability of navigation lines.

Overall, the advantages of tree-row centerline fitting lie in its clear structure and relatively low computational cost, but its performance is highly dependent on the quality of front-end trunk detection and tree-row structure recognition. When random errors exist in trunk positions, local jitter may occur in the centerline; when missing trees or curved tree rows are present, single linear fitting may cause deviations in path direction; and when curves overfit noisy points, unnecessary bending or curvature fluctuations may appear in the reference path. Therefore, tree-row centerline generation should not only satisfy spatial position accuracy but also ensure temporal continuity and curvature smoothness to prevent reference path jitter from further amplifying path-tracking control errors. However, centerline fitting may retain systematic upstream bias or introduce additional deviations when geometric association is unstable or smoothing is excessive. Therefore, the generated reference path should be evaluated using lateral position, tangential direction, fitting residual, frame-to-frame continuity, and update latency, rather than only by the accuracy of the upstream detector. A systematic centerline shift primarily biases the lateral-error input, whereas an incorrect path direction primarily biases the heading-error input [80,129,130].

#### 3.3.2. Local Path Generation Methods Based on Traversable Areas

Local path generation methods based on traversable areas no longer assume that vehicles always travel along the geometric centerline of tree rows; instead, they comprehensively consider factors such as traversable areas, corridor boundaries, obstacle positions, vehicle dimensions, implement width, and safety distance to generate locally trackable paths that satisfy environmental constraints [131,132]. Compared with tree-row centerline fitting methods, these methods place greater emphasis on the matching relationship between the actual safe traversable space of the vehicle and its motion constraints. Common methods include local searches based on cost maps, sampling-based planning, artificial potential field methods, the dynamic window approach (DWA), RRT, Hybrid A*, and spline optimization. These methods are more suitable for hilly orchard scenarios with irregular tree-row structures, complex obstacle distributions, or locally constrained corridors [133].

In research on orchard mobile robots, local path generation is often used in combination with traversable-area recognition, local map construction, and obstacle-avoidance constraints. Jiang et al. [134] proposed a hybrid path-planning algorithm based on an improved D* Lite algorithm for orchard robot path planning, demonstrating the application value of graph-search methods for path replanning and obstacle-avoidance navigation in complex orchard environments. Qu et al. [135] constructed an orchard inspection robot navigation system based on the Robot Operating System (ROS), integrating LiDAR-based SLAM with the Hybrid A*–DWA navigation method, indicating that local path generation needs to rely simultaneously on environmental maps, vehicle localization, and motion constraints. Such studies indicate that local path planning in orchards is not merely a geometric path search but requires transforming perception maps, obstacle distributions, and vehicle kinematic constraints into executable driving paths.

In terms of traversable-area and navigation-line recognition, perception results provide fundamental inputs for local path generation. Xu et al. [67] developed an orchard navigation-line recognition method based on U-Net, showing that visual segmentation methods can extract structural information related to vehicle driving direction from complex orchard backgrounds and provide navigation priors for subsequent path generation. In contrast, local planning methods further focus on obstacle avoidance and trajectory executability. Ye et al. [136] proposed an improved kinematically constrained bidirectional RRT method for unstructured orchard environments, indicating that introducing vehicle kinematic constraints can improve path executability and navigation adaptability in orchard scenarios with irregular corridors and complex obstacle distributions. Luo et al. [137] combined an improved ant colony algorithm with DWA for the path planning of orchard mobile robots, reflecting a planning strategy that integrates global search with local dynamic obstacle avoidance.

Overall, these studies show that the role of local path generation methods lies not only in bypassing obstacles, but also in transforming traversable areas, environmental boundaries, and vehicle motion constraints into reference paths that can be tracked by controllers. Local path-planning results are not necessarily compatible with path-tracking control. If path search focuses only on obstacle avoidance and the shortest distance, it may generate paths with excessively rapid heading changes or many local polyline segments; if the cost function overemphasizes keeping away from obstacles, the path may exhibit frequent lateral offsets in narrow inter-row spaces; and if the dynamic update frequency is too high, the reference path may change continuously within the control cycle, leading to unstable control targets. Therefore, local path generation for path-tracking control should simultaneously consider path length, obstacle distance, curve continuity, slope risk, and control-input smoothness, so that the generated path can satisfy the integrated requirements of traversability, executability, and trackability.

Local planning transforms traversable-area boundaries, obstacle locations, and terrain constraints into a time-varying reference path and safety corridor. Errors in obstacle positions or corridor boundaries may shift the local path or create unnecessary detours. In addition, frequent replanning may cause discontinuities between the previous and updated paths, producing abrupt changes in the target point, lateral error, path direction, or curvature. Therefore, local path quality should also be evaluated in terms of obstacle clearance, path-switching continuity, replanning frequency, and planning latency [134,135,136,137].

#### 3.3.3. Path Smoothing, Curvature Constraints, and Reference-Speed Planning

The reference path should not only be spatially traversable, but also satisfy the requirements of path-tracking control in terms of continuity, curvature variation, and speed setting. The initial path obtained from tree-row centerline fitting, traversable-area search, or local obstacle-avoidance planning may contain polyline segments, local discontinuities or abrupt changes, or regions with curvature discontinuities. If such paths are directly used as controller inputs, they may easily lead to rapid changes in vehicle heading error and abrupt variations in steering commands. Therefore, path smoothing and curvature constraints are important steps in transforming local geometric paths into control-trackable reference paths. Common methods include polynomial curves, B-spline curves, Bézier curves, clothoid curves, and optimization-based path-smoothing methods, which are used to reduce heading discontinuities and curvature discontinuities in polyline paths. Their objective is to improve the continuity of path direction, curvature, and speed planning while maintaining path traversability [138,139,140].

**Figure 6:**
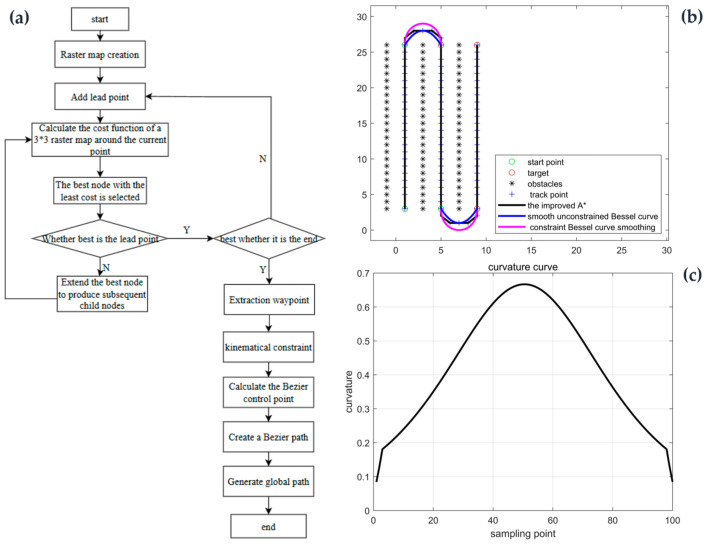
Workflow of orchard reference path generation and curvature-constrained optimization for path-tracking control: (**a**) path-planning workflow based on improved A* and multi-constraint Bézier curves; (**b**) comparison of trajectory results obtained by different path-planning methods; (**c**) curvature-continuity validation of the multi-constraint curves. (Reprinted from Ref. [143] under the Creative Commons Attribution 4.0 International license (CC BY 4.0)).

Existing studies usually introduce continuous-curvature paths, improved sampling-based planning, or curve optimization methods after local path generation to improve path trackability. Sabelhaus et al. [141] used continuous-curvature paths to generate feasible headland turning trajectories, indicating that curvature continuity is of great importance for trajectory executability in large-steering, turning, and headland operation scenarios of agricultural vehicles. Ye et al. [142] proposed a continuous bidirectional Quick-RRT* (CBQ-RRT*) algorithm for complex orchard environments, demonstrating that path-continuity constraints can improve the path quality of orchard mobile robots. Kong et al. [143] conducted path planning for an orchard fertilization robot based on multi-constraint Bézier curves, showing that curve-constrained methods can improve path smoothness and vehicle motion executability while satisfying environmental and operational constraints. To illustrate the role of curve optimization methods in orchard reference path generation, Figure 6 shows the path-planning, trajectory-comparison, and curvature-continuity validation processes based on improved A* and multi-constraint Bézier curves.

Path smoothing and curvature constraints do not merely improve geometric appearance; they directly determine the curvature and steering requirements transmitted to the controller. Insufficient smoothing may preserve local path jumps and cause abrupt steering changes. Excessive smoothing may violate obstacle-clearance or corridor constraints if boundary and obstacle information is not adequately retained during optimization. Reference-speed planning should additionally consider curvature, longitudinal and cross-slope conditions, roughness, adhesion conditions, and vehicle-stability limits. Otherwise, a geometrically feasible path may still produce excessive steering demand, slip, sideslip, or instability when executed at an inappropriate speed. However, the reviewed orchard studies more frequently report path geometry and curvature than quantified mappings from longitudinal slope, cross slope, or roughness to reference-speed limits; this remains an important evidence gap.

In summary, vehicle-state estimation provides position, heading, velocity, roll, and pitch information, whereas environmental representation and reference path construction provide reference path position, direction, curvature, speed, and safety constraints. Residual or newly introduced errors in these processes are consequently expressed as uncertainties or discontinuities in the lateral error, heading error, reference curvature, reference speed, and safety constraints used by the path-tracking controller.

### 3.4. Unified Error-Propagation Chain from Observation to Path-Tracking Control

Based on the evidence reviewed in Section 2 and the transformation processes discussed in Section 3.1, Section 3.2 and Section 3.3, this review adopts the “observation errors–vehicle-state and environmental-representation errors–reference path and controller-input errors–closed-loop responses” chain as a review-based analytical structure rather than as a standardized model already established for hilly orchard navigation.

GNSS position errors, IMU heading drift, wheel-odometry errors, visual navigation-line errors, LiDAR registration errors, and depth-measurement errors constitute the principal upstream uncertainties. Through fusion localization and environmental-structure representation, these uncertainties are retained or transformed into errors in vehicle position, heading, velocity, trunk coordinates, tree-row directions, corridor boundaries, obstacle positions, and local terrain constraints.

During reference path and controller-input construction, vehicle-state and environmental-representation errors are further converted into biases or discontinuities in lateral error, heading error, reference path position, path direction, reference curvature, reference speed, and safety constraints. Vehicle position bias and centerline-position error jointly affect lateral-error construction, whereas vehicle-heading error and path-direction error jointly affect heading-error construction. Discontinuous boundaries, unstable centerline fitting, and local-map drift may generate path jumps or curvature fluctuations, while obstacle-distance and corridor-boundary errors may result in insufficient or overly conservative safety constraints.

The closed-loop effects of these errors depend on controller sensitivity, vehicle dynamics, terrain conditions, processing delay, and actuator performance. Fusion, filtering, path smoothing, and robust control may attenuate upstream errors, whereas slope-induced slip, cross-slope-induced sideslip, actuator saturation, and rapidly changing reference paths may amplify their closed-loop effects, leading to tracking bias, steering oscillation, unnecessary detours, or safety risks. Table 2 summarizes the principal transformations, and Section 4 further examines how different controllers respond to these uncertain inputs.

## 4. Path-Tracking Control Methods and Robustness-Enhancement Strategies Under Disturbances in Hilly Orchards

Path-tracking control is a key closed-loop execution component of autonomous navigation systems and directly determines whether agricultural machinery can travel stably, safely, and continuously along the reference path. Complex environmental disturbances in hilly orchards can affect sensor observations, perception and localization, and reference path generation, and the resulting errors are ultimately transmitted to the controller input in the form of lateral error, heading error, curvature variation, and speed constraints, thereby affecting path-tracking accuracy and driving stability [144,145].

Therefore, this section analyzes the response characteristics and robustness-enhancement strategies of different path-tracking control methods in disturbed hilly orchard environments in three categories: geometry- and kinematics-based control, feedback regulation and model optimization control, and robust adaptive and intelligent control. Following the unified error-propagation chain established in Section 3.4, the controllers reviewed in this section are evaluated not only according to their nominal tracking accuracy but also according to their sensitivity to uncertainty in vehicle-state feedback, reference path position and direction, curvature, reference speed, safety constraints, vehicle–ground interaction, and actuator response. Direct field comparisons conducted under quantified hilly orchard slopes remain limited. Therefore, evidence from regular orchards, agricultural fields, and transferable off-road environments is retained with its original test context, and the reported results are not used for a direct ranking of controller performance.

### 4.1. Path-Tracking Control Methods Based on Geometric and Kinematic Models

**Figure 7:**
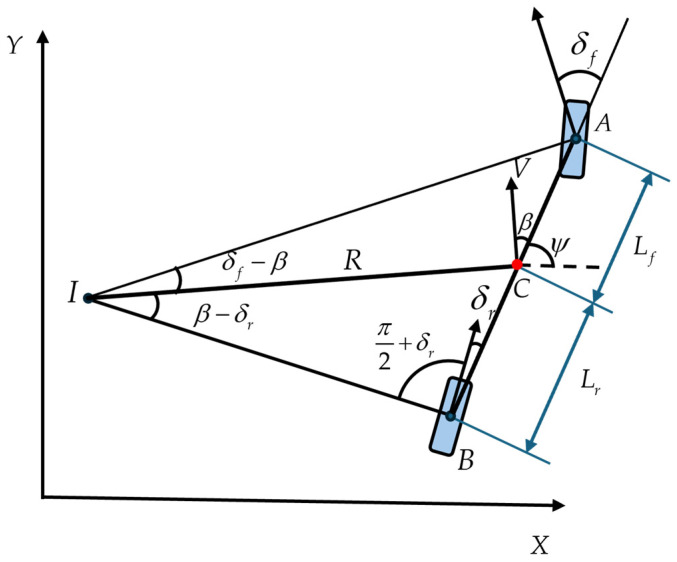
Simplified Ackermann steering vehicle model. The red dot at point *C* denotes the vehicle’s center of mass.

Geometric and kinematic models are the most commonly used modeling basis in path-tracking control of agricultural vehicles [146,147]. For low-speed wheeled orchard vehicles, a simplified Ackermann steering vehicle model is usually adopted to describe vehicle motion relationships, and control laws are constructed based on the lateral error, heading error, and path curvature of the vehicle relative to the reference path. For differential-drive or tracked platforms, similar kinematic descriptions can also be established through equivalent turning radius or angular velocity relationships. To facilitate the subsequent analysis of the control mechanisms of Pure Pursuit, Stanley, and MPC, this review first presents the simplified Ackermann steering vehicle model and the definitions of key error variables, as shown in Figure 7.

As shown in Figure 7, the front wheels of the vehicle chassis are equivalently converged at point *A*, the rear wheels are equivalently converged at point *B*, and point *C* represents the position of the vehicle’s center of mass. Here, *I_A_* and *I_B_* denote the turning radii of the front and rear axles during steering, respectively, and point *I* represents the instantaneous center of rotation during vehicle motion. *ψ* denotes the current vehicle heading angle, *β* denotes the sideslip angle, and *δ_f_* is the front-wheel steering angle. *L_f_* and *L_r_* denote the distances from the center of mass to the front and rear axles, respectively. The vehicle kinematic model is given as follows:

(1) $$V_{X}=V\cdot \cos\left(\psi +\beta \right)$$

(2) $$V_{y}=V\cdot \sin\left(\psi +\beta \right)$$

(3) $$\psi =\frac{V\cdot \cos\beta }{L_{f}+L_{r}}\cdot \left(\tan\delta_{f}-\tan\delta_{r}\right)$$

(4) $$\beta =\tan^{-1}\left(\frac{L_{f}\cdot \tan\delta_{r}+L_{r}\cdot \tan\delta_{f}}{L_{f}+L_{r}}\right)$$

Under low-speed driving conditions with a small sideslip angle, the vehicle motion process can be simplified into the following kinematic model:

(5) $$\dot{x}=V\cdot \cos\psi$$

(6) $$\dot{y}=V\cdot \sin\psi$$

(7) $$\dot{\psi }=\frac{V}{L}\cdot \tan\delta$$

where *x* and *y* denote the vehicle position, *ψ* denotes the vehicle heading angle, *V* denotes the vehicle velocity, *L* denotes the wheelbase, and *δ* denotes the equivalent front-wheel steering angle. This model has a simple structure and is suitable for analyzing the inter-row path-tracking control of low-speed orchard vehicles. However, in hilly orchards, slope-induced slip, cross-slope-induced sideslip, and variations in adhesion conditions can violate the ideal kinematic assumptions; therefore, geometric control methods usually need to be combined with parameter adjustment, curvature feedforward, sideslip compensation, or slip estimation to improve robustness [148]. The simplified model additionally assumes sufficiently accurate vehicle-state feedback, a continuous reference path, a small sideslip angle, and a sufficiently fast steering response. Consequently, the position, heading, path-direction, and curvature errors discussed in Section 3.4 enter geometric controllers through the relative target-point position, lateral error, heading error, and desired curvature. Vehicle slip, sideslip, and actuator delay further cause the actual motion response to deviate from the steering action predicted by the ideal kinematic model.

#### 4.1.1. Pure Pursuit Control

**Figure 8:**
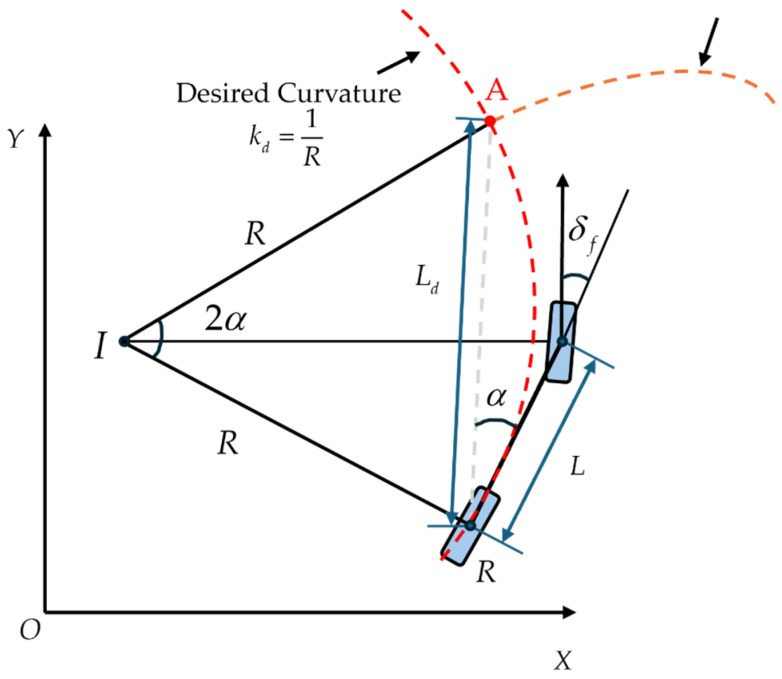
Geometric principle diagram of Pure Pursuit path-tracking control.

Pure Pursuit control is a typical geometric path-tracking method based on a look-ahead point. This method does not rely on a complex vehicle dynamic model; instead, it selects a look-ahead point on the reference path and calculates the steering command according to the geometric relationship among the current vehicle position, vehicle heading, and the look-ahead point [149]. For low-speed orchard mobile robots and agricultural vehicles, Pure Pursuit control features a simple structure, strong real-time performance, and convenient engineering implementation, and is therefore commonly used in inter-row navigation and local path-tracking tasks. Its geometric principle is shown in Figure 8.

As shown in Figure 8, under the simplified Ackermann steering model, Pure Pursuit control assumes that the vehicle moves toward the look-ahead point *A* along a circular arc, and its geometric relationship can be expressed as follows:

(8) $$R=\frac{L_{d}}{2\sin\alpha }$$

(9) $$k_{d}=\frac{1}{R}=\frac{2\sin\alpha }{L_{d}}$$

(10) $$\delta =\arctan\left(L\cdot k_{d}\right)=\arctan\left(\frac{2L\cdot \sin\alpha }{L_{d}}\right)$$

where *R* denotes the radius of the circular arc corresponding to the vehicle’s motion toward the look-ahead point, *L_d_* denotes the distance between the vehicle reference point and the look-ahead point *A*, *k_d_* denotes the desired tracking curvature determined by the geometric relationship of the look-ahead point, *α* denotes the angle between the current vehicle heading and the line connecting the vehicle to the look-ahead point, *L* denotes the vehicle’s wheelbase, and *δ* denotes the equivalent front-wheel steering angle of the Ackermann steering vehicle.

In field path-tracking tests on a crawler orchard sprayer, Wang et al. reported a mean absolute lateral error of 2.15 cm and a maximum deviation of 4.08 cm while using an adaptive look-ahead controller. In the autonomous navigation experiments, the maximum deviation and mean absolute error were 5.78 cm and 2.69 cm, respectively, and 97.32% and 100% of the deviations were within ±5 cm and ±10 cm [150]. Therefore, under complex orchard and uneven ground conditions, dynamically adjusting look-ahead parameters according to path morphology, vehicle speed, and tracking error is an important strategy for enhancing the robustness of Pure Pursuit control. From the perspective of error propagation, Pure Pursuit is directly affected by uncertainties in vehicle position, heading, reference path geometry, and look-ahead point selection. A vehicle-position bias or centerline shift changes the relative location of the look-ahead point, whereas path-direction and curvature fluctuations alter the target angle and desired curvature calculated by the controller. Moreover, slope-induced slip or track slip violates the assumed circular-arc motion, causing the executed vehicle trajectory to deviate from the geometrically predicted trajectory. These uncertainties may therefore be transformed into steering-command fluctuations, tracking lag, or persistent lateral deviation.

In existing studies, dynamic look-ahead and slip compensation have been used to improve the adaptability of the traditional Pure Pursuit method under complex ground conditions. Dynamic look-ahead methods usually adjust the look-ahead distance according to vehicle speed, path curvature, lateral error, and heading error, thereby balancing driving stability on straight segments and tracking accuracy on curved segments. Wang et al. [151] proposed a Pure Pursuit path-tracking method based on a self-adjusting look-ahead distance for the field navigation of high-clearance sprayers. By establishing a Pure Pursuit kinematic model for agricultural machinery and using an evaluation function to determine the optimal look-ahead distance within the look-ahead region, the method achieved dynamic optimization of look-ahead parameters. Yang et al. [152] improved the Pure Pursuit algorithm from the perspective of optimal target-point determination by simulating driver look-ahead behavior and searching for the optimal target point with the objective of minimizing lateral error and heading error, thereby improving path-tracking accuracy in the automatic navigation of agricultural machinery. Garrow et al. [153] improved the Pure Pursuit control method from the perspective of path-curvature sensitivity by incorporating speed, understeering characteristics, and path-curvature compensation into the target-point tracking process, providing a methodological reference for look-ahead parameter adjustment on paths with varying curvature. To address the susceptibility of tracked agricultural vehicles to slip under complex ground conditions, Liu et al. [56] estimated the left and right track slip ratios and corrected the look-ahead-point position online. Compared with the traditional fuzzy Pure Pursuit method, the proposed approach reduced the maximum lateral deviation by 78.1% and 63.1% at 0.35 and 0.75 m/s, respectively, while the corresponding average deviations were reduced by 50.6% and 57.6%. Çiloğlu et al. [154] further applied online slip estimation to the improvement of the Pure Pursuit algorithm for off-road tracked vehicles, indicating that slip compensation can reduce the deviation between the actual vehicle motion response and the ideal kinematic model.

Collectively, the reviewed studies indicate that robustness-enhancement strategies for Pure Pursuit mainly address two propagation links: instability in the look-ahead target caused by speed and reference path variations, and kinematic model mismatch caused by slip or sideslip. Pure Pursuit remains attractive for low-speed orchard platforms because of its low computational cost and ease of implementation. However, its performance in hilly orchards depends strongly on the continuity of the reference path, adaptive look-ahead selection, and reliable slip or sideslip compensation. Direct field evidence under quantified longitudinal and cross-slope conditions nevertheless remains limited.

#### 4.1.2. Stanley Control

**Figure 9:**
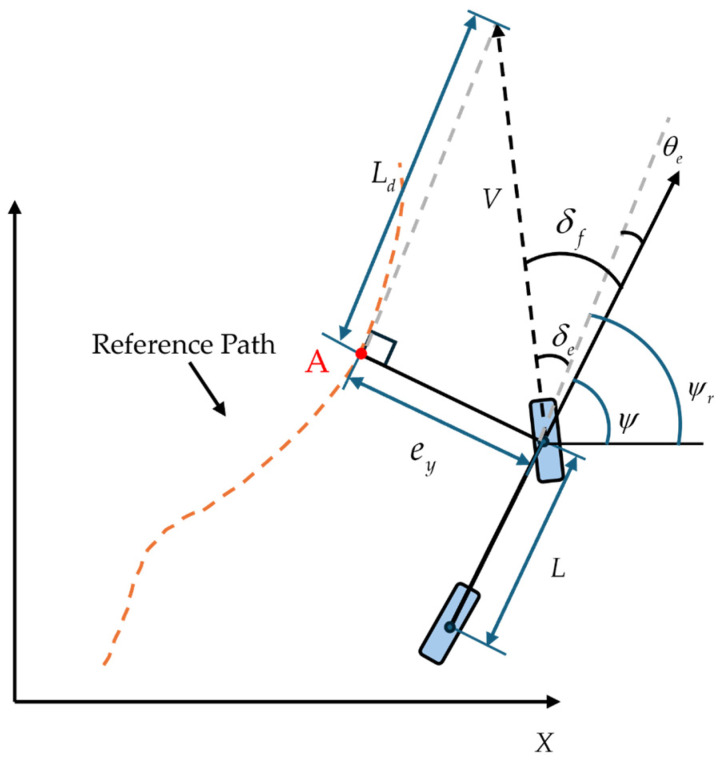
Geometric principle diagram of Stanley path-tracking control.

Stanley control is a typical geometric path-tracking control method based on lateral error and heading error. It usually takes the front-axle center as the vehicle reference point and directly generates the front-wheel steering command by calculating the lateral error from the vehicle front-axle center to the nearest point on the reference path, as well as the heading error between the current vehicle heading and the tangential direction of the reference path [155]. This method has a simple structure and a clear physical interpretation of the error terms. Therefore, it has good engineering applicability in the path-tracking control of orchard mobile robots. Its geometric principle is shown in Figure 9.

As shown in Figure 9, Stanley control first searches for the matching point *A* on the reference path that is closest to the vehicle front-axle center and takes the tangential direction of the path at this point as the desired heading. The normal distance from the vehicle front-axle center to the reference path is defined as the lateral error *e_y_* and the angle between the vehicle heading and the tangential direction of the reference path is defined as the heading error *e_ψ_*, corresponding to *θ_e_* in the figure. The controller corrects the vehicle driving direction through the heading-error term and reduces the vehicle’s lateral deviation from the reference path through the lateral-error term.

The basic error definitions and the front-wheel steering control law of Stanley control can be expressed as follows:

(11) $$e_{\psi }=\psi_{r}-\psi$$

(12) $$\delta_{e}=\arctan\left(\frac{k\cdot e_{y}}{V}\right)$$

(13) $$\delta_{f}=e_{\psi }+\delta_{e}$$

where *e_ψ_* denotes the heading error, *ψ_r_* denotes the tangential direction angle at the matching point on the reference path, *ψ* denotes the current vehicle heading angle, *e_y_* denotes the lateral error of the vehicle front-axle center relative to the reference path, *k* denotes the lateral-error gain, *V* denotes the vehicle’s traveling speed, *δ_e_* denotes the steering correction term generated by the lateral error, and *δ_f_* denotes the equivalent front-wheel steering angle of the Ackermann steering vehicle.

The limitations of Stanley control in hilly orchards are mainly reflected in its sensitivity to lateral error, heading error, and velocity estimation. When localization noise is large or the tangential direction of the reference path is unstable, the controller may generate excessive steering; when the vehicle travels at low speed, the lateral-error term is easily amplified, leading to oscillations in the steering command; and when cross-slope-induced sideslip or wheel–ground slip occurs, the actual motion direction of the vehicle may be inconsistent with the vehicle body heading, causing deviations in steering correction based on geometric errors [156]. Because the lateral and heading errors used by Stanley control are jointly constructed from estimated vehicle states and reference path geometry, the controller cannot distinguish whether an error fluctuation originates from localization or path extraction. Both sources appear as changes in the steering-correction terms. This direct dependence explains why localization jumps, unstable path tangents, and low-speed noise can produce pronounced steering fluctuations.

Recent studies have improved Stanley control through low-speed gain limitation, heading-angle filtering, speed-adaptive gain, curvature feedforward, sideslip-angle compensation, and slip compensation. These improvements aim to enhance its path-tracking stability under complex ground conditions. To address the susceptibility of agricultural tractors to sideslip on uneven ground, Wang et al. [157] proposed an improved Stanley path-tracking control method considering sideslip compensation and combined it with sliding mode control (SMC) to enhance the heading correction capability of the vehicle under lateral disturbances, thereby improving the stability and disturbance rejection of path tracking for agricultural vehicles. Hoffmann et al. [158] applied Stanley control to the trajectory tracking of off-road autonomous vehicles and considered factors such as tire dynamics and steering actuators, indicating that this type of geometric tracking method has potential adaptability to unpaved ground and complex terrain. Sun et al. [159] combined particle swarm optimization (PSO) with fuzzy Stanley control and addressed the limitations associated with fixed parameters and poor environmental adaptability of traditional Stanley control by optimizing control gains and fuzzy rules, thereby enhancing the control accuracy and stability of agricultural machinery during whole-field path tracking. In a field test of a tractor automatic-navigation system, Cui et al. used fuzzy logic to adjust the Stanley gain according to the tracking error. At a traveling speed of 1 m/s, the maximum and average lateral errors on a straight path were 10 cm and 5.2 cm, representing reductions of 16.7% and 10.3% compared with the conventional Stanley method. During whole-field navigation, the maximum lateral error was reduced from 34 cm to 27 cm [160]. Seiffer et al. [161] considered system delay, path-curvature feedforward, and a previewed feedforward reference point in Stanley control. Validation on a demonstrator vehicle showed that the improved method reduced the root-mean-square cross-track error from 0.11 m to 0.03 m on a dynamic circuit, illustrating the importance of delay compensation when the calculated steering command cannot be executed instantaneously.

Overall, Stanley control is suitable for low-speed agricultural vehicles when lateral and heading errors can be calculated continuously and the path tangent is sufficiently stable. Its principal vulnerabilities in hilly orchards are low-speed gain amplification, sideslip-induced inconsistency between body heading and actual motion direction, reference path direction fluctuations, and actuator delay. Accordingly, speed-adaptive gain, heading filtering, curvature feedforward, sideslip estimation, and delay compensation are the most relevant robustness-enhancement strategies. However, direct comparisons under quantified hilly orchard slopes and identical vehicle platforms remain scarce.

### 4.2. Path-Tracking Control Methods Based on Feedback Regulation and Model Optimization

Feedback-regulation and model-optimization methods differ from geometric controllers in how they process the controller-input errors constructed in Section 3. Proportional–integral–derivative (PID) and fuzzy controllers generate corrective actions mainly from the current and accumulated tracking errors, whereas MPC predicts future vehicle responses using vehicle states, reference paths and associated speed profiles, and constraint information. Consequently, the former are particularly sensitive to the quality and temporal continuity of error signals, while the latter are additionally affected by state-estimation uncertainty, reference-trajectory quality, prediction-model mismatch, constraint errors, and computational delay.

#### 4.2.1. PID and Fuzzy Control

In path-tracking control of agricultural vehicles, PID control is one of the earliest and most mature feedback-control methods used in engineering applications [162]. Compared with geometric control methods, PID control does not directly rely on a vehicle kinematic model but generates the steering command according to the tracking error between the actual vehicle trajectory and the reference path, and continuously corrects the vehicle’s motion state through closed-loop feedback. Its simple structure and relatively low computational cost make it widely applicable to low-speed agricultural and orchard mobile platforms [163].

The continuous form of PID control can be expressed as follows:

(14) $$u\left(t\right)=K_{p}\cdot e\left(t\right)+K_{i}\cdot \int_{0}^{t}e\left(t\right)\cdot dt+K_{d}\frac{de\left(t\right)}{dt}$$

where *e*(*t*) denotes the path-tracking error signal, which can be composed of the lateral error *e_y_*, the heading error *e_ψ_*, or a weighted combination of both; *K_p_*, *K_i_*, and *K_d_* denote the proportional, integral, and derivative coefficients, respectively. The proportional term is used to improve the error response speed, the integral term is used to eliminate steady-state error, and the derivative term is used to predict the error variation trend and suppress overshoot. From the perspective of error propagation, PID performance depends directly on the accuracy and continuity of the lateral- or heading-error signal. A persistent localization or reference path bias may accumulate in the integral term and may cause overshoot or integral windup when the actuator is saturated. In contrast, abrupt localization jumps, frame-to-frame path jitter, or high-frequency heading noise may be amplified by the derivative term, resulting in steering-command fluctuations. Excessive proportional gain may accelerate error correction but may also increase sensitivity to measurement noise and actuator delay.

**Figure 10:**
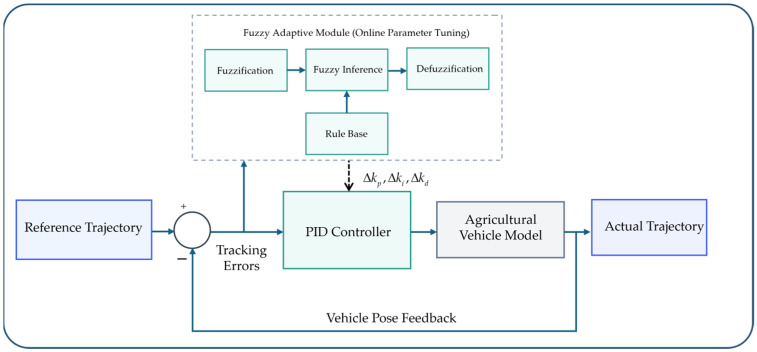
Schematic diagram of closed-loop path-tracking control using PID control and fuzzy PID control.

To improve the adaptability of PID controllers to nonlinear disturbances and changing operating conditions, fuzzy control is often combined with PID control to form a fuzzy PID control structure. Its basic idea is to use the error and the rate of change in the error as inputs for fuzzy inference to adjust PID parameters online or directly correct the control output [160,164,165]. Depending on the selected membership functions and fuzzy rules, the controller gains or steering corrections can be adjusted according to the magnitude and rate of change in the tracking error. Such adjustment may strengthen the response when deviations increase and reduce excessive correction near the reference path. However, its performance remains dependent on the design of the fuzzy rules, input scaling factors, operating speed, and the reliability of the measured error signals [164,165,166]. The closed-loop control structures of PID and fuzzy PID path tracking are shown in Figure 10.

Existing improvements in PID and fuzzy control have mainly focused on automatic parameter tuning, variable-universe fuzzy inference, steering compensation, and integration with slip-aware control. Hailu et al. [167] used PSO to tune a nonlinear fuzzy PID controller and compared it with fuzzy PID and conventional PID controllers for a differential-drive plantation robot. The study was primarily based on MATLAB/Simulink (The MathWorks, Inc., Natick, MA, USA; software versions not reported in Ref. [167]; MATLAB website: https://www.mathworks.com/products/matlab.html; accessed on 4 August 2026; Simulink website: https://www.mathworks.com/products/simulink.html; accessed on 4 August 2026) trajectory simulations and therefore provides methodological evidence for parameter optimization rather than direct hilly orchard field evidence. For single-track agricultural machinery operating under variable curvature, uneven terrain, and track-slip conditions, Liu et al. [168] combined a segmented preview model, variable-universe fuzzy control, and PSO-based support vector regression (SVR) steering compensation. Compared with fixed-preview-distance and fixed-universe fuzzy methods, the proposed method reduced the average number of steering actions per control cycle by 30.19% and 18.23%, respectively, and reduced the average lateral error by 34.29% and 46.96%, respectively. In an orchard mowing robot study, Li et al. [55] combined cascaded predictive path tracking with slip-ratio-based fuzzy control, PID drive control, and a tire-dynamics model. Field experiments maintained average lateral and longitudinal errors within 0.05 m and 0.04 m, respectively. These values represent the integrated performance of predictive path tracking and fuzzy–PID anti-slip drive control, rather than the performance of a standalone PID controller. Ma et al. [169] further combined visual navigation-line extraction with fuzzy steering control for low-speed navigation of a goji-berry orchard harvesting robot, illustrating the coupling between perception-derived error signals and fuzzy feedback control.

Overall, PID and fuzzy controllers remain attractive for low-speed agricultural platforms because of their relatively simple structure and limited computational demand. Their robustness mainly depends on the quality of the tracking-error signal, gain adaptation, anti-windup treatment, noise filtering, and compensation for slip or actuator delay. However, fixed or empirically tuned parameters are difficult to maintain across large variations in speed, path curvature, adhesion, and payload, and direct quantitative validation under explicitly sloped orchard conditions remains limited.

#### 4.2.2. MPC and Its Improvement Strategies

**Figure 11:**
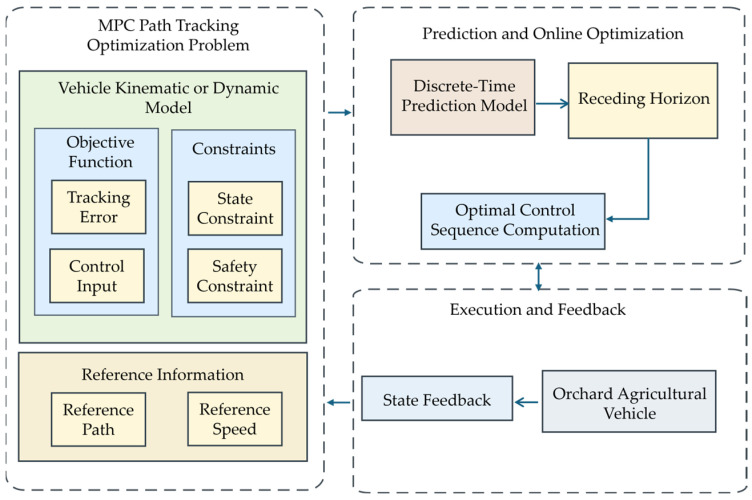
Schematic diagram of MPC-based receding-horizon optimization control for path tracking.

MPC is a path-tracking control method based on a prediction model and a receding-horizon optimization mechanism. Its core advantage lies in its ability to simultaneously consider tracking errors, control-input variations, vehicle motion constraints, and actuator constraints within a finite prediction horizon, solve an optimization problem at each control cycle, and execute the optimal control action at the current time step [170,171,172,173]. Therefore, MPC is particularly suitable for orchard path-tracking tasks involving constraints, curvature variations, and multi-objective optimization requirements. Its receding-horizon optimization control structure is shown in Figure 11.

MPC usually establishes the predictive relationship based on a discrete linear state-space model, whose basic form can be expressed as follows:

(15) $$x_{k+1}=A\cdot x_{k}+B\cdot u_{k}$$

The corresponding finite-horizon optimization objective function can be written as follows:

(16) $$\min J={\sum_{i=1}^{N_{p}}\left\|x_{k+i}-x_{r,k+i}\right\|}_{Q}^{2}+{\sum_{i=0}^{N_{c}-1}\left\|\Delta u_{k+i}\right\|}_{R}^{2}$$

where *x_k_* denotes the vehicle state or error-state vector at the kth sampling instant, which may consist of variables such as vehicle position, heading angle, velocity, lateral error *e_y_*, and heading error *e_ψ_*; *u_k_* denotes the control input to the vehicle model, usually including the front-wheel steering angle, steering-angle increment, or speed control command; and *A* and *B* denote the state matrix and input matrix of the discrete linear prediction model, respectively. *N_p_* denotes the prediction horizon, *x_r,k+i_* denotes the reference state at the *i*th step within the prediction horizon, *Q* denotes the state-error weighting matrix used to adjust the weights of state variables such as lateral error, heading error, and velocity error, *N_c_* denotes the control horizon, and *R* denotes the control-input variation weighting matrix used to suppress abrupt steering changes and control oscillations. From the perspective of the propagation chain established in Section 3.4, MPC receives multiple upstream variables simultaneously and is therefore sensitive to uncertainty at several levels. Vehicle position, heading, and velocity errors bias the initial state of the prediction model; discontinuities in reference path position, direction, or curvature distort the predicted tracking target; and errors in obstacle boundaries, permissible speed, or stability constraints may lead to unsafe or excessively conservative optimization results. Processing and actuator delays additionally reduce the consistency between the predicted and actual vehicle responses.

In hilly orchards, prediction-model mismatch is mainly caused by unmodeled longitudinal slip, cross-slope-induced sideslip, changing adhesion conditions, terrain-induced attitude variation, and actuator dynamics. These factors may cause the vehicle response predicted within the optimization horizon to differ from the actual response. MPC performance also depends on the continuity of state estimates and reference variables supplied by the upstream navigation modules. Therefore, an accurate vehicle model alone is insufficient; state-estimation reliability, reference path continuity, constraint quality, optimization time, and actuator execution capability jointly determine the closed-loop performance of MPC. The limited computational resources of small orchard platforms are an additional constraint on nonlinear or robust MPC deployment.

Existing MPC improvements can be broadly grouped into reducing model dependence, coordinating steering and actuator responses, optimizing controller weights, improving nonlinear vehicle modeling, handling bounded disturbances, and coordinating state estimation with control. To reduce dependence on an explicitly identified vehicle model, Cheng et al. [174] incorporated PID terms into model-free adaptive predictive control. For a four-wheel-independent-drive agricultural vehicle, Liu et al. [171] combined MPC with direct-yaw-moment control and reported steady-state errors below 0.22% for the steering angle, 0.17% for the yaw moment, and 1% for vehicle speed. In vehicle experiments, the actual turning radius was 9.1 m, with a maximum error of 0.55% at 1 m/s when the desired steering angle was 5°, whereas a minimum turning radius of 1.51 m was obtained with a maximum error of 6.6% at 0.5 m/s when the steering angle was 30°. These results characterize steering and vehicle-motion coordination rather than lateral path-tracking accuracy. From the perspective of terrain disturbance, Wang et al. [173] developed a data-driven tube MPC method for autonomous tractor navigation under cross-slope conditions, providing directly relevant methodological evidence for slope-disturbance prediction and robust constraint design.

More direct path-tracking evidence has been reported for articulated and tracked agricultural vehicles. Xu et al. [172] used particle swarm optimization to adjust the weighting matrix of an MPC controller for an articulated tractor. In simulation, the improved method reduced the maximum path deviation by 40% and shortened the convergence time by 37.5% compared with conventional MPC. In orchard real-vehicle tests at 3.6 km/h, the lateral-error ranges of conventional MPC, improved MPC, and PID control were −0.10–0.25 m, −0.08–0.10 m, and −0.20–0.40 m, respectively, whereas their heading-error ranges were −2–2°, −2–1°, and −3–3°, respectively. Zeng et al. [175] further incorporated vehicle roll and pitch, online track-slip estimation, and curvature-feedforward compensation into nonlinear model predictive control (NMPC) for a tracked agricultural vehicle under uneven-terrain conditions. In simulation, the proposed method reduced the mean and standard deviation of the lateral tracking deviation by 30.28% and 32.46%, respectively, and reduced the corresponding mean and standard deviation of the heading deviation by 37.27% and 35.05% compared with conventional NMPC.

Other studies have emphasized field implementation, real-time optimization, bounded uncertainty, and state-estimation–control coordination. Backman et al. [176] applied NMPC to a tractor–trailer system and validated the method using real agricultural machinery in field environments, demonstrating the feasibility of considering tractor–implement kinematics and path-tracking objectives simultaneously within the prediction horizon. Kraus et al. [177] combined moving-horizon estimation (MHE) with NMPC to estimate vehicle orientation and slip-related parameters on a wet and uneven grass field. With code-generated optimization, the reported feedback computation time was approximately 0.6–1.6 ms, indicating the real-time potential of coordinated estimation and predictive control. Jeong et al. [178] introduced tube-based robust MPC for autonomous articulated road vehicles to limit the effects of bounded model uncertainty and external disturbances. Because the vehicle platform and validation environment differed from agricultural field operations, this study is used as transferable methodological evidence rather than direct agricultural path-tracking evidence.

Overall, the reviewed MPC improvements act on different links of the error-propagation chain. Model-free predictive control reduces dependence on an explicitly identified vehicle model; adaptive weight optimization changes the controller response to lateral- and heading-error inputs; nonlinear modeling and slip estimation reduce prediction-model mismatch; moving-horizon estimation improves the consistency of the vehicle states supplied to the controller; and tube-based robust MPC limits the effects of bounded model and external disturbances. Constraint-aware reference path and speed-profile construction further reduce conflicts among path feasibility, vehicle-motion limits, and actuator constraints. MPC is advantageous when vehicle states, reference paths and associated speed profiles, and safety or actuator constraints can be provided with sufficient accuracy and continuity. However, biased state estimates, discontinuous reference paths, uncertain terrain constraints, actuator delay, and excessive optimization time may still degrade its closed-loop performance. Direct comparisons conducted on the same agricultural platform under quantified longitudinal and cross-slope conditions remain limited.

### 4.3. Robust and Intelligent Control Methods for Uncertain Disturbances

Robust and intelligent control methods address uncertainty through different mechanisms. SMC explicitly suppresses bounded model uncertainty and external disturbances through sliding-surface design and switching or continuous robust control actions. In contrast, reinforcement learning (RL) and end-to-end (E2E) methods learn control policies or intermediate navigation outputs from data, thereby reducing reliance on manually designed models or rules. However, their responses to propagated perception, localization, and reference path errors depend on disturbance bounds, observation quality, training-data coverage, reward design, and safety-supervision mechanisms.

#### 4.3.1. SMC and Its Fixed-Time Convergence Improvements

**Figure 12:**
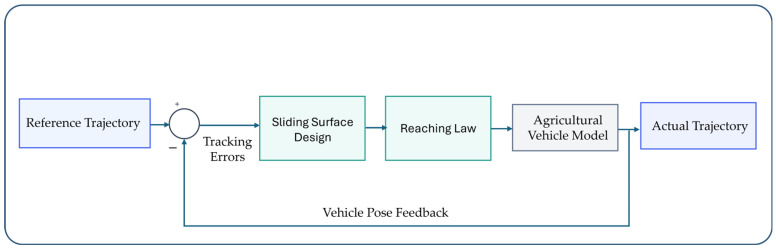
Schematic diagram of SMC for path tracking.

SMC is a typical robust nonlinear control method whose basic principle is to design a sliding surface and a robust control law so that the system state reaches and remains near the sliding surface [179,180,181,182]. Because SMC can suppress bounded model uncertainty and external disturbances, it has potential applicability to agricultural path-tracking problems involving slip, sideslip, adhesion variation, and unmodeled vehicle–ground interactions. However, direct field validation under quantified hilly orchard terrain remains limited. Its basic control structure is illustrated in Figure 12.

For illustration, conventional SMC can construct a sliding surface based on the path-tracking error, *e*, as follows:

(17) $$s=\dot{e}+\lambda e$$

The reaching law can be expressed as follows:

(18) $$\dot{s}=-ks-\eta \cdot \mathrm{sgn}\left(s\right)$$

Accordingly, the SMC law is usually composed of an equivalent control term and a switching control term:

(19) $$u=u_{eq}+u_{sw}$$

To reduce control chattering caused by the traditional sign function, the saturation function or boundary-layer method is often used in engineering applications to replace the sign function, namely,

(20) $$\mathrm{sgn}\left(s\right)\to sat\left(\frac{s}{\phi }\right)$$

where *s* denotes the sliding variable or sliding surface; *e* denotes the path-tracking error, which can be composed of the lateral error *e_y_* and the heading error *e_ψ_*; *ė* denotes the error rate of change; *λ*, *k*, and *η* are positive control parameters that affect the shape of the sliding surface, the error reaching speed, and disturbance rejection capability, respectively; *u* denotes the control input to the vehicle model, representing the front-wheel steering angle, steering-angle increment, or angular velocity control command; *u_eq_* denotes the equivalent control term used to compensate for system motion under the nominal model; *u_sw_* denotes the switching control term used to suppress external disturbances and model uncertainties; and sgn(⋅) denotes the sign function, which generates a switching control action according to the positive or negative direction of the sliding variable *s* so that the system state converges to *s* = 0 from both sides of the sliding surface. The function sat(⋅) denotes the saturation function, which is used to replace the sign function to construct a boundary layer, thereby reducing control chattering caused by discontinuous switching. *ϕ* denotes the boundary-layer thickness.

From the perspective of error propagation, the sliding variable is constructed from the lateral error, heading error, and their rates of change, and therefore contains uncertainties originating from both vehicle-state estimation and reference path construction. A persistent localization or centerline bias shifts the equilibrium represented by the sliding surface, whereas high-frequency position or heading noise may be amplified by a large switching gain or by the differentiation of the error signal. Abrupt reference path direction or curvature changes may produce rapid variations in the sliding variable, while actuator delay and saturation may prevent the commanded robust action from being executed as assumed.

The main advantage of SMC is its ability to maintain tracking stability in the presence of bounded disturbances and model uncertainty. Nevertheless, conventional SMC has several limitations for agricultural vehicles. Discontinuous switching may cause steering chatter and frequent actuator actions, whereas an excessively thick boundary layer may reduce steady-state accuracy. The required switching gain usually depends on the assumed disturbance bound, which is difficult to determine when slip, sideslip, and soil adhesion vary continuously. Moreover, localization noise, path jitter, and error differentiation may generate fluctuations in the sliding variable. Therefore, disturbance rejection should be balanced against measurement-noise sensitivity, steering smoothness, actuator bandwidth, and steady-state accuracy.

Existing SMC improvements mainly include adaptive gain adjustment, reaching-law optimization, disturbance-observer compensation, super-twisting structures, prescribed-performance constraints, and finite- or fixed-time convergence. In field tests of an unmanned rice transplanter on slippery paddy soil, Li et al. [183] used a radial basis function (RBF) neural-network-based adaptive SMC method. Compared with conventional sliding-mode control, the maximum and average absolute lateral deviations decreased from 17.5 cm to 9.3 cm and from 9.1 cm to 3.2 cm, respectively. The maximum heading deviation decreased from 46.7° to 3.1°, while the average absolute heading deviation decreased from 10.7° to 1.3°. These results provide direct agricultural field evidence under sideslip and soil-disturbance conditions, although the test environment was a paddy field rather than a hilly orchard.

Other studies have focused on reducing chattering and compensating for unknown disturbances. Ji et al. [184] combined a modified super-twisting controller with a disturbance observer for unmanned agricultural tractors. Zhang et al. [185] integrated nonsingular fast terminal SMC with disturbance estimation to improve finite-time error convergence, whereas Qiao et al. [186] optimized the reaching law for harvesting-robot control. Yang et al. [187] combined fast super-twisting control with an anti-peaking extended-state observer to reduce transient observer effects and improve disturbance compensation. These studies demonstrate different mechanisms for improving convergence and disturbance rejection, but their platforms, disturbance settings, and evaluation indices differ and should not be directly ranked.

Fixed-time and prescribed-performance approaches further attempt to constrain both convergence time and transient error. Sun et al. [188,189] combined generalized super-twisting or nonsingular terminal sliding-mode structures with slip consideration and adaptive disturbance observation, so that the error-convergence time was not directly determined by the initial tracking deviation. More recently, Li et al. [180] combined prescribed-performance constraints, a generalized extended-state observer, and switched sliding-mode control. In high-fidelity co-simulations, the standard deviation of the preview error was reduced by up to 92.52%, 84.33%, and 80.44% compared with PID, MPC, and conventional observer-based SMC, respectively. These values are simulation-based comparative evidence rather than hilly orchard field results.

Overall, SMC is particularly relevant when the dominant closed-loop uncertainty arises from slip, sideslip, model mismatch, or external disturbances. Adaptive gains and disturbance observers reduce dependence on accurately known disturbance bounds, while super-twisting and continuous control laws help suppress chattering. Fixed-time and prescribed-performance methods additionally constrain convergence behavior. However, SMC does not eliminate upstream localization or reference path errors; high-frequency error signals may still be amplified, and strong switching actions may exceed actuator bandwidth. Direct validation on the same platform under quantified longitudinal slopes, cross slopes, localization degradation, and path disturbances remains scarce.

#### 4.3.2. RL and E2E Control

RL and E2E methods provide data-driven alternatives for agricultural navigation and path-tracking control. RL learns a control or decision policy through the interaction with a training environment, whereas E2E methods learn mappings from sensor observations or vehicle states to intermediate waypoints, local trajectories, or direct control commands. These approaches may reduce dependence on manually tuned controller parameters or explicitly designed perception features, but they do not eliminate uncertainty. Instead, perception noise, localization bias, reference path discontinuities, slip, and sideslip are embedded in the observation distribution and learned policy [190].

From the perspective of error propagation, an RL controller may interpret localization jumps, unstable path curvature, or perception failures as valid state changes unless these conditions are represented during training or detected by an independent safety monitor. Reward functions that emphasize only lateral-error reduction may produce aggressive steering actions or sacrifice control smoothness and obstacle clearance. In addition, policies trained within limited ranges of speed, terrain, adhesion, and sensor noise may not generalize reliably to hilly orchard conditions outside the training distribution. Epistemic uncertainty estimation and safety intervention can therefore be used to identify low-confidence actions and provide risk-aware control supervision [191].

Existing agricultural RL studies have explored model-free path tracking, curvature-aware policy learning, and safety-constrained navigation. Zhang et al. [192] trained a double deep Q-network (Double DQN) path-tracking controller in simulation and evaluated it on a grass field with multiple sharp turns. Compared with Pure Pursuit control, the learned controller reduced corner overshoot and settling time at relatively high speeds, although a slightly longer rise time and a higher steady-state error were also reported. Li et al. [193] developed an improved deep Q-network (DQN) framework for agricultural-machinery path tracking, providing additional agricultural methodological evidence for learning control policies from vehicle-state and tracking-error information. Zhang et al. [194] subsequently incorporated the average curvature of the forward reference path into a DQN controller. Under the reported test conditions, the average tracking errors were 0.023–0.036 m on soft and flat ground and 0.029–0.037 m on hard and uneven ground, outperforming the corresponding Pure Pursuit results. For orchard tractor navigation, Hu et al. [195] combined unmanned aerial vehicle (UAV) photogrammetry with safe RL and maintained the average lateral deviation within 0.13 m in the reported orchard experiments.

Uncertainty-aware safe RL provides another mechanism for limiting unsafe learned actions. In road-vehicle research, Zhang et al. [191] introduced epistemic uncertainty estimation into a safe RL framework and used the quantified uncertainty to adjust safety constraints and support intervention during deployment. Because this study was conducted on road vehicles, it provides transferable methodological evidence for uncertainty-aware safety supervision rather than direct hilly orchard field evidence.

E2E control should be distinguished from learning-assisted intermediate navigation. A strict E2E controller directly maps raw sensor observations to steering, velocity, or other control commands. Bakken et al. [196] used a deep convolutional neural network to predict steering angles for crop-row following directly from red–green–blue (RGB) images; however, the reported field evaluation was preliminary and open-loop rather than a complete closed-loop field validation. In contrast, Jang et al. [197] developed a long short-term memory (LSTM)-based imitation learning method for curvature-adaptive waypoint generation using Global Positioning System (GPS) trajectories collected from real orchard operations. Because its output consists of intermediate waypoints rather than direct steering commands, this method is more accurately classified as a learning-based reference construction. Ren et al. [198] integrated orchard perception, dynamic RANSAC path construction, and RL-based path tracking within a modular system and therefore represents a hybrid perception–planning–control framework rather than a strict E2E mapping. In simulated outdoor agricultural navigation, Khanzada et al. [199] reported a normalized performance score of 0.92 for deep-neural-network (DNN)-based behavior cloning in the precision and autonomy scenarios, whereas real-time appearance-based mapping (RTAB-Map) achieved the highest score of 0.96 when navigation speed was prioritized. These simulation results illustrate that learning-based and conventional navigation methods exhibit different accuracy, autonomy, and efficiency trade-offs.

Overall, current RL and E2E studies provide promising agricultural and orchard evidence, but they do not yet support independent deployment or direct performance ranking against conventional controllers in quantified hilly orchard terrain. Their more realistic near-term roles include waypoint generation, reference path correction, gain or weight adaptation, disturbance estimation, and residual control within a model-based safety framework. Independent safety monitors should retain the authority to limit speed and steering, reject low-confidence actions, trigger replanning, or switch to a verified conventional controller. Hybrid architectures that combine learning-based adaptation with MPC or other model-based constraints are therefore more consistent with the present evidence than completely model-free closed-loop deployment.

### 4.4. Summary

This section reviews geometric and kinematic control, feedback-regulation and model-optimization control, and robust, adaptive, and learning-based control for agricultural machinery operating under disturbances relevant to hilly orchards. As summarized in Table 3, Pure Pursuit and Stanley control have simple structures and low computational demands, but their performance depends strongly on reliable position and heading feedback, continuous reference path geometry, appropriate parameter adjustment, and compensation for slip, sideslip, and actuator delays. PID and fuzzy control are convenient for engineering implementation, although fixed or empirically tuned parameters are difficult to generalize across changes in speed, adhesion, payload, and terrain. MPC provides a systematic means of incorporating vehicle states, reference paths and associated speed profiles, actuator limits, speed constraints, and safety margins, but it relies on prediction-model validity, state-estimation continuity, constraint accuracy, and real-time computational capability. SMC is suitable for suppressing bounded disturbances and model uncertainty, whereas chattering, noise amplification, and actuator-bandwidth limitations remain important concerns. RL and E2E methods offer adaptation potential, but the present evidence more strongly supports their use for waypoint generation, reference path correction, parameter adaptation, disturbance estimation, or residual control within a model-based safety framework rather than independent closed-loop deployment.

The suitability of a controller, therefore, depends less on its nominal algorithm category than on whether its structure matches the dominant error-propagation pathway and the available sensing, computation, and actuation conditions. Position and centerline biases mainly disturb geometric target-point relationships and lateral-error feedback; heading and path-direction errors directly affect steering correction; curvature discontinuities and frequent replanning generate abrupt control demands; and slip, sideslip, model mismatch, and actuator delay weaken the consistency between the calculated control action and the actual vehicle response. Robustness enhancement should therefore combine controller-level adaptation with reliable state estimation, continuous reference path construction, disturbance estimation, actuator compensation, and explicit safety constraints.

Quantitative evidence must be interpreted separately from the method-level synthesis. Because the reviewed studies differ in vehicle platform, operating speed, terrain condition, path geometry, localization system, payload, disturbance intensity, and error definition, their reported values cannot be normalized into a reliable cross-study ranking. Table 4 is therefore presented as an evidence map that retains the original controller, vehicle platform, validation context, performance metric, and evidence category of each study. The reported values indicate the order of magnitude achieved under the original experimental conditions rather than a generally achievable accuracy for the corresponding controller category.

## 5. Current Challenges and Future Trends

The evidence reviewed in Section 2, Section 3 and Section 4 indicates that the principal limitation of autonomous navigation in hilly orchards is no longer the absence of individual perception, localization, planning, or control algorithms. Instead, the remaining challenges lie in the reliability of front-end observations under compound disturbances, the lack of quantitative mappings from upstream uncertainty to controller inputs and closed-loop responses, and the difficulty in implementing integrated navigation systems under vehicle-specific hardware and real-time constraints. Future research should therefore focus on three interconnected priorities: uncertainty-aware perception and localization with failure awareness, quantitative error-propagation modeling and closed-loop sensitivity analysis, and hardware-aware perception–planning–control integration supported by virtual–real validation and independent safety supervision.

### 5.1. Reliability Challenges of Perception and Localization in Complex Hilly Orchard Environments

In hilly orchards, factors such as canopy occlusion, terrain undulation, cross-slope-induced sideslip, variations in ground adhesion, and irregular tree-row structures continuously degrade the quality of multi-source observations, resulting in concurrent GNSS position jumps, IMU drift, slip-induced wheel-odometry errors, unstable visual features, and LiDAR-registration degradation. Existing studies mostly evaluate the performance of front-end modules using localization RMSE, detection accuracy, or mapping accuracy; however, these average accuracy metrics are insufficient to reflect the effects of low-confidence scenarios, local sensor failures, and conflicts among multi-source observations on subsequent path-tracking controller inputs. Therefore, the key challenge of perception and localization in hilly orchards is no longer merely to improve the average accuracy of a single sensor or algorithm, but rather to identify observation degradation states, quantify the confidence of state estimation, and transmit this confidence to reference path generation and the controller.

Future technical routes should further shift from “high-precision localization” toward “reliable localization and failure awareness.” Multi-source fusion systems should output not only vehicle position, heading, velocity, and attitude, but also localization covariance, sensor health status, observation confidence, scene degradation types, and failure warning information. Adaptive multi-sensor fusion should combine reliability estimation with abnormal-observation rejection so that downstream modules receive state estimates accompanied by interpretable and actionable confidence information. When observation confidence decreases or sensor conflicts persist, these uncertainty indicators should support concrete downstream actions, such as adaptive sensor weighting, temporary retention of the last reliable reference path, reference-speed reduction, enlargement of the safety margin, sensor switching, or entry into a controlled degraded mode.

### 5.2. Error Propagation Modeling for Closed-Loop Control

Current research on agricultural machinery navigation in hilly orchards still commonly adopts a module-by-module evaluation approach: the perception module focuses on detection accuracy, the localization module focuses on pose error, the path-planning module focuses on path length and smoothness, and the control module focuses on lateral error or tracking RMSE. However, in practical closed-loop systems, front-end perception and localization errors do not remain within a single module, but propagate progressively through vehicle state estimation, environmental-structure representation, reference path generation, and controller-input construction, and are further manifested as lateral-error bias, heading-error fluctuation, abrupt changes in reference curvature, unreasonable reference-speed assignment, and mis-specification or violation of safety constraints. The lack of error propagation modeling may cause the controller to passively respond only to tracking errors that have already formed, making it difficult to use upstream uncertainty estimates for timely predictive mitigation. It should be emphasized that the four-layer propagation chain summarized in Section 3.4 is a review-based analytical framework for organizing the available evidence, rather than a standardized quantitative model that has already been established and validated for hilly orchard navigation.

In future studies, controller-oriented error-propagation modeling should begin by defining a common set of measurable variables at each stage of the navigation chain. These variables should include raw observation errors and availability indicators, vehicle position and heading errors, tree-row and corridor-geometry deviations, reference path position and direction errors, curvature fluctuations, reference-speed changes, safety-margin errors, actuator responses, and final lateral and heading tracking errors. Probabilistic, sensitivity-based, and data-driven models can be used to characterize how upstream uncertainties affect lateral error, heading error, reference curvature, speed, and safety constraints. Establishing these mappings requires the time-synchronized recording of raw sensor observations, ground-truth vehicle pose, intermediate perception outputs, generated reference paths, controller inputs, actuator commands, and actual vehicle responses. Controlled experiments should separately introduce GNSS jumps, IMU drift, wheel or track slip, tree-row detection offsets, point-cloud registration errors, processing delays, and path discontinuities, and should then examine their combined effects under different longitudinal and cross-slope angles, speeds, payloads, adhesion conditions, and path curvatures.

The resulting models should identify not only whether an upstream error affects a downstream variable, but also its sensitivity, time delay, persistence, amplification or attenuation, dominant operating conditions, and the threshold at which tracking stability or safety begins to deteriorate. At the control level, error covariance, path confidence, and failure flags should be used to adjust controller gains, prediction horizons, constraint tightening, reference speed, and safety margins before large tracking errors develop.

### 5.3. Hardware-Aware Closed-Loop Integration and Virtual–Real Validation

Path-tracking control for agricultural machinery in hilly orchards should shift from “single-module performance improvement” toward the integrated collaborative design of “perception and localization–reference path–robust control.” As shown in Figure 1, the proposed framework comprises four functional layers—observation, state estimation and environmental-structure perception, reference path planning and controller-input construction, and robust control—which are connected through forward error propagation and supervisory feedback. In this framework, the observation layer characterizes environmental and vehicle–ground disturbances in complex hilly orchards; the state-estimation and environmental-structure-perception layer generates vehicle pose estimates, environmental-structure representations, and perception-reliability information; the reference-path-planning and controller-input-construction layer generates trackable reference paths and controller inputs while characterizing the propagation of upstream errors; and the robust-control layer performs path-tracking control and evaluates closed-loop performance. Safety supervision and degraded operation close the loop by using perception reliability, reference path quality, tracking performance, vehicle attitude, and actuator states to trigger sensor-weight adjustment, path updating or replanning, reference-speed reduction, safety-margin adjustment, a controlled stop or manual takeover. Implementation should distinguish between wheeled or Ackermann-steered machinery and tracked or differential-drive platforms. The former is mainly constrained by steering dead zones, backlash, steering-rate limits, actuator delay, and cross-slope-induced sideslip, whereas the latter is more strongly affected by asymmetric track slip, soil deformation, motor saturation, and differential-speed allocation. Actuator-state feedback should be incorporated into closed-loop performance assessment and safety supervision to distinguish perception- or path-related errors from incomplete command execution and vehicle–ground interaction disturbances, and to support controller adjustment or degraded operation.

Model-driven constraints can provide interpretable safety boundaries, while data-driven components can be restricted to uncertainty estimation, disturbance compensation, parameter adaptation, or residual control. Digital twins and virtual–real fusion should be developed through a staged validation route. Field measurements should first be used to parameterize orchard geometry, longitudinal and cross slopes, surface roughness, ground adhesion, vehicle dynamics, sensor degradation, communication delay, and actuator response. The resulting virtual environment should support the controllable injection of GNSS jumps, IMU bias, visual occlusion, LiDAR registration degradation, depth-data loss, synchronization errors, slip, sideslip, and actuator saturation, followed successively by software-in-the-loop, processor- or hardware-in-the-loop, and controlled field validation. Meanwhile, a unified evaluation system for hilly orchards should be established, incorporating perception confidence, reference path quality, slip and sideslip estimation accuracy, actuator-command execution, tracking accuracy and control smoothness, degraded-operation effectiveness, operational safety, and long-term autonomous-operation capabilities, thereby promoting research from local algorithmic improvements toward reliable system-level applications.

## 6. Conclusions

This review examines the propagation of perception and localization errors and robust path-tracking control for agricultural machinery in hilly orchards. From the perspective of closed-loop control requirements, it systematically analyzes the coupling among sensor observation, vehicle pose estimation, environmental-structure perception, reference path generation, and path-tracking control. Unlike reviews that classify studies only according to sensors, planning methods, or control algorithms, this review emphasizes that front-end perception and localization errors are progressively transformed along the autonomous navigation chain into deviations in controller inputs, such as lateral error, heading error, reference curvature, reference speed, and safety constraints, thereby affecting path-tracking accuracy, control smoothness, and operational safety. On this basis, the reviewed evidence is organized into a four-layer, controller-oriented framework linking observation errors, state and environmental uncertainty, controller-input disturbances, and robust closed-loop responses.

The synthesis indicates that upstream errors do not have uniform downstream effects. Positioning jumps and centerline offsets mainly bias lateral-error construction; heading drift and path-direction errors disturb heading feedback; path discontinuities and terrain-representation errors affect reference curvature, reference speed, and safety constraints; and slip, cross-slope-induced sideslip, actuator delay, and saturation may further amplify these disturbances into tracking bias, oscillation, response lag, and reduced safety margins. Accordingly, controller suitability depends on the dominant error-propagation pathway, vehicle platform, terrain and adhesion conditions, reference path quality, and available sensing, computation, and actuation capabilities, rather than on nominal algorithm accuracy alone.

Several limitations of this review should be acknowledged. First, direct field evidence from quantitatively characterized hilly orchards remains limited. Second, the included studies are highly heterogeneous in terms of vehicle platforms, terrain characteristics, sensor configurations, operating speeds, evaluation metrics, and validation conditions, which limits direct quantitative comparison across studies. Third, no formal study-quality or risk-of-bias assessment was conducted; therefore, differences in evidence quality should be considered when interpreting the synthesized findings. Consequently, the integrated framework presented in this review should be regarded as a synthesis-derived design direction rather than an experimentally validated complete perception–planning–control architecture. Future research should prioritize synchronized full-chain datasets, quantitative error-propagation and closed-loop sensitivity models, uncertainty-aware reference path and speed adaptation, slip- and sideslip-aware robust control, and staged virtual–real and field validation under longitudinal slopes, cross slopes, canopy occlusion, and variable adhesion conditions.

## Figures and Tables

**Table 1:** Primary navigation measurements, representative reported performance metrics, and major error sources of key observation sources in hilly orchards.

Observation Source	Primary Navigation Measurements	Representative Reported Metric and Error Magnitude	Main Error Source	References
GNSS	Global position observation	Moving-position RMSE: 0.152 m in an open field, 0.182 m in an orchard, and 1.130 m in a mountainous agricultural environment; mean canopy RTK error: approximately 0.07–0.18 m	Canopy occlusion, multipath effects, loss of RTK fixed solutions	[42,50]
IMU	Attitude, angular velocity, acceleration, and short-term motion propagation	Heading-error RMS below 1.5° under the reported attitude conditions; orchard BDS/IMU heading-error bound approximately 2°	Bias drift, vibration, attitude coupling	[51,52]
Wheel odometry	Wheel angular velocity, vehicle speed, and odometry increments	No generalizable absolute error magnitude; error accumulates with distance and increases under slip	Slip and adhesion variation	[53,54,55,56]
Vision sensors	Semantic information such as tree rows, trunks, boundaries, and traversable areas	Navigation-line distance error approximately 0.0503–0.056 m; binocular lateral and heading errors 0.020 m and 0.57°	Illumination, shadows, occlusion, and weeds	[65,66,67,68,69]
LiDAR	Geometric structures of trunks, ground surfaces, obstacles, and boundaries	Integrated odometry translation/rotation errors: 0.068 m/0.856°; navigation lateral MAE 0.0183 m	Sparse point clouds, occlusion, and attitude disturbances	[76,79,80,81]
Depth perception sensors	Short-range depth and spatial structure	Trunk-location error 0.009 m at 2.8 m; effective field interval approximately 0.160–1.400 m for RealSense D435i	Strong illumination, edge distortion, and low texture	[45,82,85,87]

**Table 2:** Transformation of perception and localization outputs into controller-related variables and associated error-propagation forms.

Transformation Stage	Representative Methods	Controller-Related Output	Main Error-Propagation Form	References
Vehicle-state estimation and multi-source fusion localization	GNSS/INS, GNSS/IMU/wheel-odometry fusion, GNSS/IMU/LiDAR fusion, LiDAR–inertial odometry, and VIO	Vehicle position, heading angle, velocity, roll, and pitch; state-reliability indicators where available	Residual position, heading, and velocity errors affect lateral-error and heading-error construction, coordinate transformation, look-ahead adjustment, and motion prediction	[96,97,98,99]
Tree-row and trunk structure recognition	Object detection, semantic segmentation, point-cloud clustering, and geometric fitting	Trunk coordinates, left–right tree-row associations, tree-row direction, and candidate inter-row center points	Missed or false trunk detection, incorrect row association, and geometric-fitting errors produce centerline-position and path-direction deviations	[68,69,81,105,106]
Inter-row corridor and traversable-area recognition	Ground and semantic segmentation, depth-based boundary extraction, occupancy grids, and semantic cost maps	Left and right corridor boundaries, available corridor width, vehicle-clearance margin, and traversability confidence	Boundary misclassification alters the feasible corridor and safety margin, resulting in local-path offsets, unnecessary detours, or insufficient obstacle clearance	[65,82,87,112,113,114]
Obstacle, boundary, and three-dimensional terrain representation	Obstacle detection, semantic point clouds, occupancy and elevation maps, and traversability analysis	Obstacle positions, longitudinal slope, cross-slope inclination, roughness, traversability, and speed or stability constraints	Obstacle-position and terrain-representation errors affect safety boundaries, permissible speed, lateral-stability margins, and non-traversable-region identification	[118,119,120,121,122,123,124]
Tree-row centerline fitting and reference path generation	Line and polynomial fitting, RANSAC, B-spline, Bézier curves, and multi-frame updating	Reference path position, tangential direction, and path-continuity information	Systematic centerline shifts bias the lateral-error input, whereas direction fluctuations and discontinuous fitting affect heading error and reference path continuity	[70,80,127,129,130]
Local path and safety-corridor generation	A*, D* Lite, RRT, Hybrid A*, DWA, and local trajectory optimization	Local reference path, safety corridor, obstacle clearance, and updated tracking target	Obstacle or boundary uncertainty shifts the local path; frequent replanning or discontinuous path switching may cause abrupt changes in target point, lateral error, direction, and curvature	[134,135,136,137]
Path smoothing, curvature constraints, and reference-speed planning	Curve smoothing, curvature and minimum-turning-radius constraints, Bézier or continuous-curvature optimization, and speed planning	Reference curvature, reference speed, and vehicle-feasibility constraints	Insufficient smoothing preserves curvature discontinuities, whereas excessive smoothing may violate clearance constraints; inappropriate reference speed may increase tracking lag, lateral acceleration, slip, sideslip, or instability risk.	[138,139,140,141,142,143]

**Table 3:** Controller mechanisms, propagated-error sensitivities, robustness-enhancement strategies, and engineering applicability of path-tracking methods for agricultural machinery in hilly orchards.

Control Method	Core Mechanism and Controller Inputs	Dominant Propagated-Error Sensitivity and Closed-Loop Effects	Typical Robustness-Enhancement Strategies	Engineering Applicability and Remaining Limitations	Representative References
Pure Pursuit	Geometric look-ahead point tracking using vehicle position, heading, speed, and reference path geometry	Positioning jumps and path discontinuities shift the look-ahead point, while slip causes tracking lag, steering fluctuations, and lateral bias	Adaptive look-ahead, speed adaptation, curvature compensation, slip-aware target-point correction	Low computational demand and convenient implementation; strongly dependent on path continuity and reliable slip estimation	[54,148,149,150,151,152]
Stanley	Direct feedback of lateral and heading errors	Heading noise, positioning jumps, and cross-slope-induced sideslips disturb error feedback; actuator delay may cause oscillation and response lag	Speed-adaptive gain, heading filtering, curvature feedforward, sideslip estimation, delayed compensation	Suitable for low-speed agricultural vehicles with stable path direction; sensitive to low-speed noise and actuator delay	[155,156,157,158,159]
PID and fuzzy control	Feedback based on current, accumulated, and changing tracking errors	Persistent bias may cause integral accumulation, whereas positioning jumps and terrain-induced high-frequency disturbances may amplify overshoot and steering fluctuations	Fuzzy tuning, PSO optimization, gain scheduling, filtering, anti-windup, slip compensation	Simple structure and low computation; parameter generalization remains difficult	[53,165,166,167]
MPC	Prediction and optimization based on vehicle states, reference paths and associated speed profiles, models, and constraints	State jumps, reference path discontinuities, and terrain-dependent model mismatch reduce prediction accuracy and may produce unsafe or conservative control actions	NMPC, tube MPC, adaptive weights, slip and slope estimation, MHE, constraint tightening, lightweight solvers	Suitable for multivariable and constrained control; requires reliable states, models, constraints, and real-time computation	[168,169,170,171,172,173,174,175,176,177]
SMC	Robust control based on a sliding surface and disturbance rejection	Slip, sideslip, terrain disturbances, and actuator uncertainty affect the sliding dynamics, while measurement noise and abrupt state changes may aggravate chattering	Adaptive gain, disturbance observer, super-twisting control, continuous reaching law, fixed-time and prescribed-performance control	Relevant to slip, sideslip, and model uncertainty; chattering and noise sensitivity remain concerns	[177,178,179,180,181,182,183,184,185,186,187]
RL and E2E control	Learning mappings from vehicle states, tracking errors, reference path information, or sensor observations to waypoints, trajectories, or control actions	Perception failures, positioning jumps, and unseen terrain conditions may cause distribution shift and unstable or low-confidence control outputs	Safe RL, uncertainty estimation, imitation learning, residual control, model-based constraints, safety monitors, and controller fallback	Adaptable to complex environments and suitable for waypoint generation, reference correction, or residual control; limited by data requirements, interpretability, safety guarantees, sim-to-real transfer, and insufficient field validation	[188,189,190,191,192,193,194,195,196,197]

**Table 4:** Representative quantitative path-tracking studies, reported performance metrics, and evidence categories.

Control Method	Vehicle/Platform and Validation Context	Reported Metric and Quantitative Result	Evidence Category	Reference
Adaptive Pure Pursuit	Crawler orchard sprayer; field test at Hou Bai Farm; straight-path tracking at 0.7 m/s	Lateral-position MAE: 0.0215 m; maximum deviation: 0.0408 m; SD: 0.0114 m	Orchard field test	Wang et al. [150]
Slip-aware Pure Pursuit	Tracked differential-drive agricultural vehicle; soft cultivated farmland; loop path with turning radii of 2.5–5.0 m; tests at 0.35 and 0.75 m/s; three trials per condition	At 0.35 m/s, maximum deviation: 0.082–0.097 m and mean deviation: 0.039–0.041 m. At 0.75 m/s, maximum deviation: 0.106–0.112 m and mean deviation: 0.049–0.053 m. Maximum-error reductions of 78.1% and 63.1% were reported relative to fuzzy Pure Pursuit.	Agricultural field test	Liu et al. [56]
Fuzzy Stanley	Dongfanghong 1104 tractor; agricultural field; straight-path test at 1 m/s and whole-field path containing straight and curved segments	Straight path: maximum/average lateral error = 0.100/0.052 m; SD = 0.034 m. Whole-field path: maximum/average lateral error = 0.270/0.081 m; SD = 0.094 m.	Agricultural field test	Cui et al. [160]
Variable-universe fuzzy control	Tracked agricultural machine; uneven open farmland with weeds; 0.56 m/s; three repeated trials	Mean lateral error: 0.0498 ± 0.0016 m; lateral-error SD: 0.0601 ± 0.0009 m. Heading-angle MAE decreased from 10.06° without compensation to 3.78° with compensation	Agricultural field test	Liu et al. [168]
PSO-MPC	Articulated tractor; orchard real-vehicle test at 1.0 m/s; complementary CarSim–Simulink simulation with a soil-adhesion coefficient of 0.4	Field test: lateral-error range = −0.08 to 0.10 m; heading-error range = −2° to 1°. Simulation: lateral RMSE = 0.06 m; maximum lateral deviation = 0.10 m.	Ordinary orchard field test plus simulation	Xu et al. [172]
LSTM–Tube MPC	New Holland T1404B tractor with AllyNav TR100; real cross-slope agricultural field; simulations included constant, gradual, rapid, and step cross-slope disturbances up to ±15°	Field test: lateral RMSE = 0.010 m; maximum lateral error = 0.040 m; MAE = 0.005 m; steady-state error = 0.022 m. Simulated lateral RMSE = 0.004–0.023 m across the four slope scenarios.	Explicit cross-slope agricultural test, non-orchard	Wang et al. [173]
Slip-aware NMPC	Tracked agricultural vehicle; orchard terrain with local undulations, shallow ruts, and mild slopes; no-load, 50 kg, and 100 kg conditions	Mean lateral deviation = 0.0843/0.1486/0.1284 m and maximum lateral deviation = 0.2492/0.4621/0.3636 m under no-load, 50 kg, and 100 kg conditions, respectively.	Orchard with unquantified mild slopes	Zeng et al. [175]
Adaptive SMC	FLW 2ZG-6DM rice transplanter; slippery paddy field; soil unevenness below 0.05 m and mud-foot depth of approximately 0.25 m	Adaptive SMC: maximum/mean absolute lateral deviation = 0.079/0.032 m; maximum/mean absolute heading deviation = 2.7°/1.3°.	Transferable agricultural field test	Li et al. [183]
Prescribed-performance SMC	DX1204 tractor; U-shaped agricultural field path; approximately 1 m/s; initial lateral offset of approximately 0.4 m	Straight segments: maximum/minimum lateral error = 0.0745/−0.0615 m; MAE = 0.02435 m; SD = 0.02795 m. MAE and SD were reduced by 39.3% and 42.5%, respectively, relative to conventional SMC.	Agricultural field test	Zhu et al. [179]
Curvature-aware DQN	Agricultural vehicle; 0.5 m/s; sharp S-shaped paths on flat-soft and uneven-solid farmland; row intervals of 5 and 6 m	Average tracking error = 0.023–0.036 m on flat, soft ground and 0.029–0.037 m on uneven, hard ground; corresponding RMSE ranges = 0.015–0.030 m and 0.026–0.039 m.	Agricultural field test	Zhang et al. [194]
Safe RL	Compact orchard tractor; flat standardized apple orchard; U-shaped orchard path at 0.4 m/s	Single representative field trial: lateral maximum/mean/RMSE = 0.19/0.11/0.13 m; heading maximum/mean/RMSE = 5.91°/2.62°/2.73°.	Orchard integrated-system test	Hu et al. [195]

## Data Availability

No new data were created or analyzed in this study. Data sharing is not applicable to this article.
